# Deterministic Pilot Risk–Benefit Assessment of Latvian Inland Fish: Safe Weekly Consumption Guidance

**DOI:** 10.3390/foods15050901

**Published:** 2026-03-05

**Authors:** Janis Rusko, Elizabete Murniece, Santa Sibule, Ilva Lazda, Dzintars Zacs, Ruta Medne, Inese Siksna

**Affiliations:** Institute of Food Safety, Animal Health and Environment “BIOR”, Lejupes Street 3, LV-1076 Riga, Latvia; elizabete.murniece@bior.lv (E.M.); santa.sibule@bior.lv (S.S.); ilva.lazda@bior.lv (I.L.); dzintars.zacs@bior.lv (D.Z.); ruta.medne@bior.lv (R.M.); inese.siksna@bior.lv (I.S.)

**Keywords:** freshwater fish, risk–benefit assessment, methylmercury, PFAS, omega-3, EPA + DHA, safe consumption, Latvia

## Abstract

Fish consumption provides nutritional benefits but can also contribute to exposures to bioaccumulative contaminants, requiring guidance that integrates both dimensions. We conducted a deterministic pilot risk–benefit assessment of Latvian inland lake fish using pooled samples stratified by lakes and species. Risks were characterized for methylmercury, estimated from total mercury, and for Σ4 PFAS (PFOS, PFOA, PFNA, PFHxS) by calculating weekly intakes under three consumption scenarios (150, 300, and 450 g/week) for a 70 kg adult and comparing them to health-based guidance values. Benefits were quantified as weekly contributions of EPA + DHA, iodine, and protein relative to reference intakes, combined into a nutritional index and integrated with risk using a benefit–risk quotient (BRQ). The primary decision outputs were safe weekly consumption amounts (g/week) and the contaminant limiting factor. Across lake-species groups, mercury was the dominant constraint on safe consumption for most predatory fish, while PFAS limited selected groups with lower mercury burdens. EPA + DHA provided the strongest differentiating benefit signal between groups, whereas iodine contribution was limited because measurements were left-censored and constant after limit of quantification (LOQ) handling. This pilot demonstrates an interpretable framework for generating lake- and species-specific consumption guidance that can be updated as monitoring coverage expands.

## 1. Introduction

Fish and fishery products are widely recognized as components of a healthy diet, providing high-quality protein and essential micronutrients (e.g., iodine and selenium), as well as long-chain n-3 polyunsaturated fatty acids (LC n-3 PUFA), particularly eicosapentaenoic acid (EPA) and docosahexaenoic acid (DHA). In European dietary contexts, seafood contributes meaningfully to nutrient adequacy and, when replacing less-favorable animal-source foods, may support improved cardiometabolic profiles at the population level. Evidence syntheses underpinning dietary guidance generally support regular fish consumption (often framed as 1–3 servings per week), with benefits attributed both to LC n-3 PUFA intake and to substitution effects within overall dietary patterns [1,2,3]. The European Food Safety Authority (EFSA) has also evaluated the health benefits of seafood consumption in European diets, supporting its role as a nutrient-dense food group [4].

At the same time, fish can contribute to dietary exposure to environmental contaminants that bioaccumulate in aquatic food webs. Mercury is a long-standing concern because methylmercury (MeHg) biomagnifies with trophic levels; consequently, larger and predatory species tend to exhibit higher concentrations than non-predatory fish [5,6]. EFSA’s risk assessment established a tolerable weekly intake (TWI) for MeHg (1.3 µg per kg body weight) that is frequently used to support consumer advice and risk management decisions related to fish consumption [5]. This risk–benefit tension is especially relevant for vulnerable groups (e.g., women of childbearing age and children), where neurodevelopmental endpoints drive protection goals, while maintaining nutritional adequacy remains important [7].

Per- and polyfluoroalkyl substances (PFASs) have emerged as additional drivers of concern in aquatic systems, including freshwater environments. EFSA’s CONTAM Panel performed a group-based human health risk assessment for four PFASs (PFOS, PFOA, PFNA, PFHxS) and established a group TWI for their sum (Σ4 PFAS), reflecting shared effects and exposure considerations [8,9]. Empirical studies across European freshwater systems have reported PFAS occurrence in fish and highlight species- and site-specific variability linked to local contamination sources and food web structure [10,11]. Such variability complicates generic “one-size-fits-all” advice and strengthens the case for stratified assessments that are explicit about which hazards drive consumption constraints in particular lake–species combinations.

Recent studies have applied quantitative risk–benefit frameworks to fish consumption using transparent ratio-based indicators that combine contaminant risk characterization with nutritional benefit evaluation. For example, benefit–risk quotient (BRQ) approaches have been implemented for freshwater fish/crayfish consumption to integrate omega-3 benefits with contaminant exposure metrics, and similar risk–benefit ratio evaluations have been used for fish products to jointly interpret metals and nutritional attributes [12,13]. In parallel, PFAS-focused assessments highlight that even modest fish consumption can lead to exceedance of safety thresholds under current health-based guidance values, underscoring the need for stratified, context-specific decision-support outputs [14].

In the Baltic region, fish consumption advice has traditionally been shaped by contaminant profiles in specific fisheries, but inland freshwater fish can display distinct patterns of accumulation and exposure relevance. Latvia has a strong tradition of recreational and household consumption of inland fish (e.g., pike, pikeperch, bream, roach), yet recent guidance integrating both nutritional value and chemical safety into a single decision-support output has been limited. Addressing nutrients and contaminants in isolation can produce contradictory messages—for example, avoiding predatory fish to reduce MeHg exposure may also reduce EPA + DHA intake if lower-risk alternatives are not clearly identified; conversely, promoting fish consumption without lake–species nuance may increase the likelihood of exceeding health-based guidance values among frequent consumers.

Therefore, the objective of this study was to develop a deterministic pilot risk–benefit assessment (RBA) for Latvian inland lake fish using pooled samples stratified by lake and species, quantifying benefits (EPA + DHA, iodine, protein) alongside key risks (MeHg, estimated from total Hg; and Σ4 PFAS) under weekly fish consumption scenarios relevant to consumer communication. The intended output is practical: lake- and species-specific safe consumption recommendations (g/week) and identification of the limiting contaminant driver, enabling risk managers to move beyond generic messaging toward precise, evidence-based recommendations that remain straightforward to communicate. In addition, we introduce an integrated benefit–risk quotient (BRQ) to compare the overall nutrition–risk balance across fish groups, while emphasizing that consumer guidance is ultimately based on the discrete safe-consumption categories.

## 2. Materials and Methods

### 2.1. Study Area and Sampling Design

**Figure 1:**
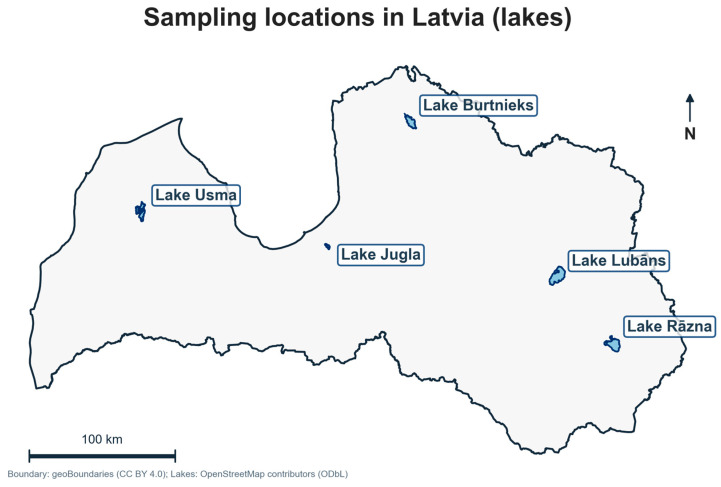
Sampling locations in Latvia (study lakes). The map shows the five inland lakes included in the pooled composite dataset (Burtnieks, Usma, Jugla, Lubāns, Rāzna).

Fish samples were collected from five major inland lakes in Latvia: Burtnieks, Jugla, Lubāns, Rāzna, and Usma as illustrated in Figure 1. An asterisk (*) denotes trophy-fish sampling in Lake Jugla (minimum length 75 cm) and is used consistently in tables and figures. This Jugla* stratum was intentionally designed to reflect an upper-end angler scenario in an urban lake, where large predatory individuals are most relevant for potential high contaminant accumulation (size/age-related bioaccumulation). In Latvia, angling rules also explicitly regulate large pike retention (one pike > 75 cm may be kept), so the trophy threshold provides a policy-relevant ‘high-end’ stratum rather than a representative sample of typical-size fish. The study targeted seven commercially and recreationally significant species: perch (*Perca fluviatilis*), pike (*Esox lucius*), tench (*Tinca tinca*), bream (*Abramis brama*), roach (*Rutilus rutilus*), pikeperch (*Sander lucioperca*), and carp (*Cyprinus carpio*).

To ensure representativeness and reduce the influence of individual variability, a composite sampling strategy was employed. In total, 460 individual fish specimens were collected across the five lakes, comprising 72 perch, 73 pike, 84 tench, 79 bream, 91 roach, and 51 pikeperch. These individuals were subsequently aggregated into pooled composites for chemical and nutrient analyses as described below. These were pooled into 134 composite samples based on species and lake of origin. The pooled composites were prepared from the above individual specimens by combining multiple fish per composite within the same lake and species stratum. Carp was included in the initial target species list; however, it was not represented in the pooled fillet/muscle composite dataset because catch rates and availability were insufficient to obtain a comparable set of specimens across lakes and sampling periods. In the study context, carp occurrence is sporadic and not representative of typical Latvian inland fisheries (e.g., possible escapee-derived presence in Lake Lubāns), and future targeted sampling would be required if carp is to be incorporated into lake-specific guidance. Because nutrient analyses are less expensive, replicate pooled composites were sometimes prepared for nutrient testing, while contaminant analyses were typically performed on a smaller number of pooled composites per stratum; therefore, the number of available pooled composites differs by analyte. Each composite sample consisted of muscle tissue from multiple individual fish of the same species and lake. Pooled composites typically consisted of 5–7 individual fish samples. Where individual sub-samples were documented, recorded pool sizes were up to 10 fish (median 9; n = 16 documented composites), noting that sub-sample documentation is incomplete for many composites.

Samples were collected during two distinct periods: Autumn (September–October 2024) and Summer (July–August 2025). While 134 pooled composites were collected in total (fillet/muscle and liver), the edible-tissue dataset comprises pooled fillet/muscle composites (n = 119 pooled composites). A predefined core nutrient panel was available for n = 67 pooled fillet/muscle composites (fat and protein), and this core panel underlies the planned/performed completeness summary. This subset covers the targeted lake–species strata included in the pooled fillet/muscle dataset across the five lakes. The final analytical coverage in the core nutrient panel were: fat (100.0%), protein (100.0%), metals (100.0%), iodine (93.7%), fatty acids (91.0%), and PFASs (83.6%) (see Table S1, Supplementary Materials; pooled sample counts by lake × species are summarized in Table S2).

### 2.2. Chemical and Nutritional Analysis

#### 2.2.1. Contaminants

Total Mercury: Total mercury (Hg) was determined using inductively coupled plasma mass spectrometry (ICP-MS) following high-pressure digestion, according to LVS EN 15763:2010 [15]. Samples were digested with nitric acid (HNO_3_) and hydrogen peroxide (H_2_O_2_) in a microwave system. Gold (Au) was added to the standard and sample solutions to stabilize mercury and minimize memory effects. The method limit of quantification (LOQ) was sufficient to detect relevant environmental levels, and measurement accuracy was verified using Certified Reference Materials (e.g., TORT-2). For risk characterization, methylmercury (MeHg) was estimated conservatively as 95% of the total mercury content (C_MeHg_ = 0.95 × C_Hg_) [5]. Methylmercury was not measured directly in the present dataset; thus, this conversion follows EFSA’s default approach for fish muscle in the absence of speciation data. Because MeHg/total Hg ratios may vary by species and environment, this assumption is treated as a pragmatic pilot approximation and is reflected in the limitations (Section 4.4).

In addition to total Hg, the standard metals panel for fish muscle included lead (Pb), cadmium (Cd), and total arsenic (As). In the pooled fillet composites used for the RBA, these analytes were below the method LOQs and therefore did not contribute meaningfully to hazard ranking in this dataset; accordingly, they were not carried forward as risk drivers in the present pilot.

Per- and Polyfluoroalkyl Substances (PFASs): The determination of PFASs (PFOS, PFOA, PFNA, and PFHxS) was performed using high-performance liquid chromatography coupled with high-resolution Orbitrap mass spectrometry (HPLC-Orbitrap-MS), using the same analytical method as described by Zacs et al. [11]. Homogenized tissue samples were extracted using alkaline digestion (0.2 M NaOH/acetonitrile) followed by solid-phase extraction (SPE) on weak anion exchange columns (Strata X-AW). Analytes were separated on a C18 column and detected in negative electrospray ionization mode using parallel reaction monitoring (PRM). Quantification was performed using isotope-dilution mass spectrometry with ^13^C-labeled internal standards (e.g., ^13^C_8_-PFOS, ^13^C_8_-PFOA) to correct for matrix effects and recovery losses.

#### 2.2.2. Nutrients

Fatty Acids (EPA + DHA): The fatty acid profile was determined by gas chromatography with flame ionization detection (GC-FID) according to the in-house method. Lipids were extracted from the fish muscle using an acetone/hexane mixture. Fatty acid methyl esters (FAMEs) were prepared via transesterification using sodium methoxide (NaOMe) and separated on a highly polar capillary column (RT-2560 or equivalent). EPA and DHA were identified by retention time comparison with a standard FAME mix (Supelco 37) and quantified as a percentage of total fatty acids, then converted to absolute concentration (mg/kg) based on the total fat content.

Iodine: Iodine was determined by ICP-MS following alkaline extraction with tetramethylammonium hydroxide (TMAH) according to the in-house method. Samples were extracted with 25% TMAH at 85 °C for 3 h. Tellurium (Te) was used as an internal standard to correct non-spectral interferences.

Protein: Crude protein content was determined using the Kjeldahl method (N × 6.25) according to standard procedures (ISO 5983-2 or equivalent) [16].

### 2.3. Risk–Benefit Assessment Strategy

**Figure 2:**
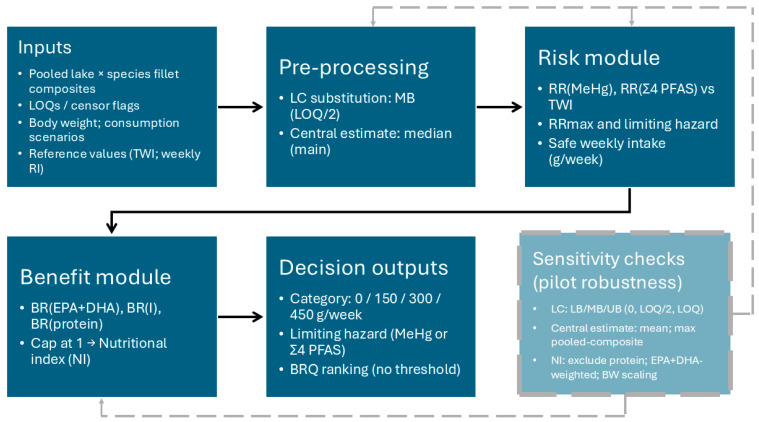
Schematic overview of the deterministic pilot RBA workflow. Pooled lake × species composites are processed using middle-bound substitution and median central estimates to derive risk ratios against TWIs (MeHg, Σ4 PFAS), safe weekly intake and limiting hazard, and nutrient benefit ratios combined into a nutritional index. Outputs are expressed as discrete safe-intake categories (0/150/300/450 g/week), limiting hazard, and BRQ as a comparative ranking indicator (no threshold implied). Targeted sensitivity checks evaluate robustness to left-censoring handling, central-estimate choice, nutritional index specification, and body weight.

A deterministic risk–benefit assessment (RBA) framework was applied to integrate nutritional benefits and chemical risks of inland lake fish consumption at the lake × species level using pooled (composite) muscle samples. The approach follows an established fish/seafood RBA practice, where scenario-based nutrient contributions and contaminant exposures are quantified on a common consumption basis and translated into decision-relevant outputs for consumer advice. Throughout the manuscript, ‘risk–benefit assessment (RBA)’ refers to the overall assessment framework, whereas ‘benefit–risk quotient (BRQ)’ refers specifically to the comparative ranking indicator derived from the nutrient and risk indices. An overview of the calculation workflow and outputs is provided in Figure 2.

#### 2.3.1. Overall Design, Scenarios and Data Handling

All calculations were performed separately for each lake × species group. For each nutrient and contaminant, the median concentration (wet weight) of pooled samples within the group was used as the central estimate, consistent with a transparent pilot decision-support design (deterministic screening). This work is intentionally presented as a partial pilot RBA, focusing on two priority hazard groups (MeHg and Σ4 PFAS) and three benefit components (EPA + DHA, iodine, protein) for which consistent occurrence data were available across lake × species strata. PFAS measurements were unavailable for two strata (Rāzna–pike and Rāzna–pikeperch); these strata were therefore assessed using the mercury-based risk component only and flagged as PFAS data gaps in the outputs (Supplementary Table S3). Because concentration data in fish often show right-skew and occasional high values (e.g., large predatory individuals), the median was selected as the default central estimate to support a robust, interpretable pilot screening comparison across lake × species strata. Upper percentiles require stable within-stratum distributions and sufficient individual-level observations; this is not available under a pooled-composite design. The median-of-pools therefore represents a central tendency for each stratum and is intended for screening-level guidance, while acknowledging that it may underestimate upper-tail exposure relevant to frequent consumers. To make this limitation explicit and quantify its practical impact on decision outputs, targeted sensitivity analyses using alternative central estimates (mean and max pooled-composite) were added (Section 2.3.6). Weekly consumption scenarios were set at 150, 300, and 450 g/week (approximately one, two, and three portions/week), and calculations assumed a 70 kg adult body weight.

Left-censored results (<LOQ) were handled using the middle-bound substitution approach (LOQ/2) in line with EFSA guidance on the management of left-censored data in dietary exposure assessment [17]. To quantify the impact of left-censoring handling where censoring was substantial (notably iodine, and selected PFAS measurements), we additionally evaluated LB (0), MB (LOQ/2), and UB (LOQ) substitution and summarized its effect on advice categories, limiting hazards, and BRQ ranking (Section 2.3.6; Supplementary Table S6). Where Σ4 PFAS (Σ4 PFAS = PFOS + PFOA + PFNA + PFHxS; sum on wet weight basis) data were not available for a given lake × species group, integrated metrics and safe-consumption estimates were derived from the available hazard information, and the corresponding outputs were clearly flagged as based on partial hazard coverage to avoid implying complete assessment for all hazards.

#### 2.3.2. Risk Characterisation

Chemical risk was characterized for methylmercury (MeHg) and Σ4 PFAS (PFOS, PFOA, PFNA, PFHxS) using health-based guidance values expressed as tolerable weekly intakes (TWI). MeHg was not measured directly and was estimated as a fixed fraction of total mercury concentration:

(1) $$C_{MeHg}=0.95\times C_{Hg}$$

where *C_Hg_* is the measured total mercury concentration (wet weight) and *C_MeHg_* is the estimated methylmercury concentration. Σ4 PFAS was calculated as the sum of PFOS, PFOA, PFNA, and PFHxS concentrations (wet weight), and group medians were used for each lake × species stratum.

For each hazard *i*, weekly intake under a given consumption scenario was calculated from the median concentration:

(2) $$Intake_{i}=C_{i}\times Cons$$

where $C_{i}\;$ is the median concentration of contaminant i in fish muscle on a wet-weight basis (e.g., Hg/MeHg in mg/kg ww; Σ4 PFAS in µg/kg ww) and *Cons* is weekly fish consumption (g/week). For intake calculations, *Cons* was converted to kg/week by dividing by 1000. Concentration units were converted as needed to match the TWI units (MeHg TWI in µg/kg bw/week; Σ4 PFAS TWI in ng/kg bw/week). Accordingly, the weekly intake per body weight was calculated as

(3) $$Intake_{i}({TWI}_{units})=C_{i}({TWI}_{mass/kg})\times \frac{Cons\left(g/week\right)}{1000}÷BW(kg)$$

with additional ×1000 or ÷1000 conversions applied when moving between mg–µg–ng.

Risk was expressed as a risk ratio (RR) relative to the TWI:

(4) $$RR_{i}=\frac{Intake_{i}}{TWI_{i}\times BW}$$

where *TWI_i_* is the tolerable weekly intake for contaminant *i* (mass/kg bw/week) and *BW* is body weight (kg). Values *RR_i_* > 1 indicate exceedance of the health-based guidance value at the specified scenario. A single conservative risk indicator per lake × species group was defined as

(5) $$RR_{max}=max(RR_{MeHg},RR_{\Sigma 4PFAS})$$

The limiting factor was identified as the hazard producing *RR_max_* (MeHg vs. Σ4 PFAS). Risk characterization followed EFSA health-based guidance values for weekly exposure. Total mercury concentrations were converted to methylmercury using the assumption MeHg = 0.95 × Hg_total, and scenario-based weekly intakes were compared to the EFSA TWI for MeHg (1.3 µg/kg bw/week) [5]. For PFASs, exposure was characterized using Σ4 PFAS (PFOS + PFOA + PFNA + PFHxS) and compared to the EFSA group TWI (4.4 ng/kg bw/week) [7].

#### 2.3.3. Benefit Characterisation and Integration

Nutritional benefits were quantified for EPA + DHA, iodine, and protein by converting median concentrations to weekly intakes under each consumption scenario and comparing these to pre-defined weekly reference intakes selected for interpretability and comparability across groups. For each component *j*, a benefit ratio (BR) was calculated:

(6) $$BR_{j}=\frac{Intake_{j}}{RI_{j}}$$

For nutrients, median concentrations were converted to weekly intakes using the same mass-balance approach:

(7) $$Intake_{j}=C_{j}\times \frac{Cons}{1000}$$

where *Intake_j_* is in mg/week (EPA+DHA), µg/week (iodine), or g/week (protein), depending on the units of *C_j_*. Benefit ratios were then computed using reference intakes expressed in matching weekly units (1750 mg/week for EPA + DHA; 1050 µg/week for iodine; 406 g/week for protein for a 70 kg adult).

To avoid dominance of the combined index by a single nutrient with very high relative contribution, benefit ratios were capped at 1:

(8) $$BR_{j,cap}=min(BR_{j},1)$$

A nutritional index (0–1 scale) was calculated as the arithmetic mean of the capped benefit ratios:

(9) $$NutritionalIndex=\frac{1}{3}\left(BR_{EPA+DHA,cap}+BR_{Iodine,cap}+BR_{Protein,cap}\right)$$

Benefit characterization used dietary reference values to express nutrient contributions on a comparable weekly basis: for EPA + DHA, an intake of 250 mg/day was applied (equivalent to 1750 mg/week) [18]; for iodine, an adequate intake of 150 µg/day for adults was applied (equivalent to 1050 µg/week) [19]; and for protein, a population reference intake of 0.83 g/kg bw/day was used (equivalent to ~58 g/day and ~406 g/week for a 70 kg adult) [20]. These reference values were used to compute nutrient contribution ratios and the integrated risk–benefit metrics described below.

Rationale for nutrient selection and weighting. The benefit component was designed as a pilot, screening-level indicator that remains interpretable and relies on analytes available across lake × species strata. EPA + DHA was selected as a fish-relevant differentiating nutrient marker, iodine as a key micronutrient with established reference intakes, and protein as a core macronutrient contribution of fish. Equal weighting of capped benefit ratios was used as a transparent default because there is no single endpoint-independent basis to prioritize these nutrients within a generic composite index without introducing value judgments. Capping each benefit ratio at 1 additionally prevents dominance by any single component. The influence of the equal-weight assumption was assessed via targeted sensitivity analyses excluding protein and applying an EPA + DHA-emphasized weighting scheme (Section 2.3.6; Supplementary Table S7).

An integrated benefit–risk quotient (BRQ) was then computed by relating nutritional benefit to the dominant chemical risk at the same scenario:

(10) $$BRQ=\frac{NutritionalIndex}{RR_{max}}$$

Higher BRQ values indicate a more favorable balance (greater nutritional contribution per unit of limiting risk) under the assumptions of this deterministic pilot. No benchmark or threshold is proposed for BRQ, and it should not be interpreted as an absolute measure of net health benefit or harm. In this framework, BRQ is used for comparative ranking/triage across lake × species strata, while consumer guidance is based on the explicit safe-consumption outputs (g/week and 0/150/300/450 category) and the limiting hazard (MeHg vs. Σ4 PFAS).

#### 2.3.4. Safe Weekly Consumption

For each lake × species group, safe weekly consumption (g/week) was defined as the maximum weekly intake that does not exceed the health-based guidance value for either hazard:

(11) $$RR_{MeHg}\leq 1\;\mathrm{and}\;RR_{\Sigma 4PFAS}\leq 1$$

Operationally, the safe amount was computed by solving the risk ratio equation for consumption for each hazard and taking the minimum:

(12) $$Cons_{safe}=min(Cons_{safe,MeHg},Cons_{safe,\Sigma 4PFAS})$$

(13) $$Cons_{safe,i}=\frac{TWI_{i}\times BW}{C_{i}}$$

For communication, *Cons_safe_* was additionally mapped to the discrete categories aligned with the three scenarios (150/300/450 g/week), while retaining continuous values for transparency.

#### 2.3.5. Uncertainty Considerations

Key uncertainties include the use of pooled samples (which smooths individual variability and may underestimate upper-tail exposures), deterministic use of medians rather than probabilistic distributions, and the influence of LOQ substitution for analytes with substantial censoring. These limitations were considered acceptable for a pilot decision-support assessment intended to identify priority lake × species combinations for targeted follow-up monitoring and to provide transparent, updateable consumption guidance.

#### 2.3.6. Sensitivity Analyses

To quantify the impact of the deterministic central-estimate choice on the primary decision outputs, all risk–benefit calculations were re-run using (i) the mean concentration (instead of the median) within each lake × species group, and (ii) the maximum pooled-composite concentration observed within each lake × species group as a conservative check. For each variant, we summarized changes in (a) safe weekly consumption (g/week), (b) the discrete communication category (0/150/300/450 g/week), and (c) the limiting hazard (MeHg vs. Σ4 PFAS). We report the number of groups that change category and/or limiting hazard relative to the main (median-based) analysis, and list the affected lake × species groups.

Left-censored sensitivity (iodine and Σ4 PFAS). Left-censored results (<LOQ) were addressed in the main analysis using middle-bound substitution (MB = LOQ/2). To quantify the impact of this choice where censoring was substantial, we additionally evaluated lower-bound (LB = 0) and upper-bound (UB = LOQ) substitution for iodine and Σ4 PFAS. For each lake × species stratum, we recomputed the iodine contribution and nutrient score, the Σ4 PFAS risk ratio, BRQ ranking, and the primary decision outputs (advice category and limiting hazard). Results are summarized in Supplementary Table S6.

Nutritional index sensitivity (weighting). To test whether BRQ ranking is sensitive to nutritional index construction, we repeated BRQ calculations for the 300 g/week scenario using (i) an index excluding protein (mean of capped EPA + DHA and iodine benefit ratios) and (ii) an EPA + DHA-emphasized weighted index (0.5 × EPA + DHA, 0.25 × iodine, 0.25 × protein; using capped benefit ratios). Rank stability relative to the main equal-weight index was quantified using Spearman correlation and by counting strata with substantial rank shifts (Supplementary Table S7).

These sensitivity analyses also provide a robustness check for BRQ ranking, which depends on the nutritional index specification and LC handling (Supplementary Tables S6 and S7).

Body weight scaling. Risk ratios were calculated using TWIs expressed per kg body weight, and the results are presented for a 70 kg adult for communication consistency. Because *RR ∝ Cons/BW*, the safe weekly consumption derived from *RR* = 1 scales linearly with body weight:

(14) $${Cons}_{safe}\left(BW\right)={Cons}_{safe}\left(70\right)\times \frac{BW}{70}$$

For example, a value of 150 g/week for a 70 kg adult corresponds to approximately 107 g/week at 50 kg and 64 g/week at 30 kg under the same assumptions.

### 2.4. Generative AI and Computational Tools

Python scripts executed within Cursor IDE (v2.2) were used for data processing, deterministic intake calculations, metric derivation, and visualization. Any AI-assisted features were limited to supporting code drafting and formatting; all outputs were reviewed and validated by the authors, who take full responsibility for the final content. Custom Python scripts used for data processing, scenario-based intake calculations, metric derivation, and figure generation are available from the authors upon request.

## 3. Results

### 3.1. Hazard Profiles and Risk Ratios

**Figure 3:**
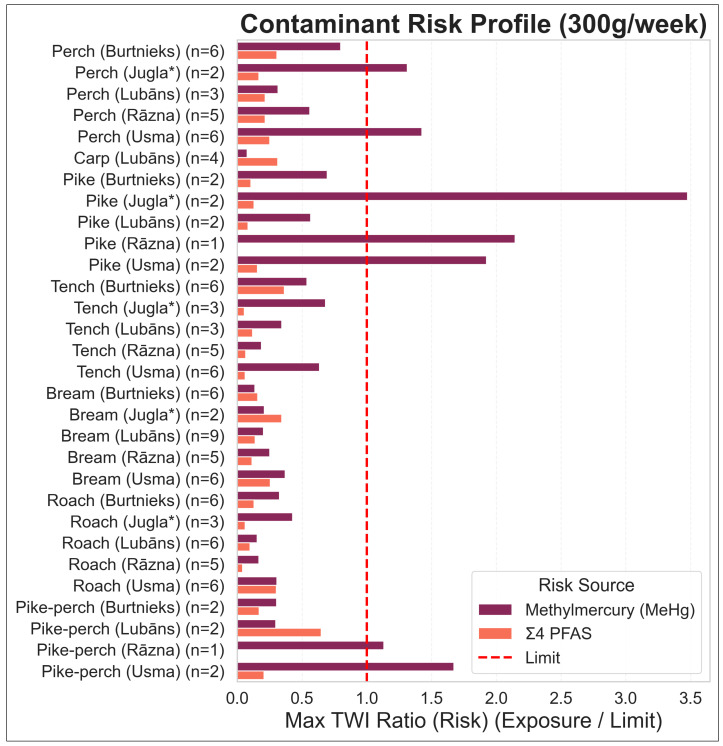
Risk drivers at the 300 g/week scenario (70 kg adult). Risk ratios (RR) for MeHg and Σ4 PFAS are shown by lake × species group, calculated against EFSA TWIs; values > 1 indicate TWI exceedance. Shading indicates the limiting hazard (higher RR) for each group. * *Jugla* indicates trophy-fish sampling in Lake Jugla (minimum fish length 75 cm).

The deterministic assessment covered 30 lake × species groups (5 lakes × 7 species, fillet tissue only). At the 300 g/week consumption scenario (approximately two fish meals per week), 7 out of 30 groups were projected to exceed at least one health-based guidance value (MeHg TWI or PFAS TWI). Across all groups, MeHg was the limiting hazard (i.e., yielded the higher risk ratio) in 25 groups, while Σ4 PFAS was limiting in 5 groups. The MeHg risk ratio at 300 g/week ranged from 0.08 (well below the TWI) up to 3.48 in the most contaminated case. In contrast, the Σ4 PFAS risk ratio (for those groups where PFAS data were available) ranged from 0.06 to 0.66, meaning that none of the measured PFAS concentrations would cause an exceedance of the PFAS TWI even at 300 g/week. Σ4 PFAS risk ratios were also robust to the LB/MB/UB substitution approach, with no resulting changes in advice categories and a limiting-hazard change observed only for a single stratum (Usma–Roach) (Supplementary Table S6). At the lake level, total mercury concentrations were highest in Jugla (particularly in the Jugla* trophy stratum), while the other lakes showed generally lower distributions across pooled composites (see the mercury-by-lake distribution figure). This indicates that both lake context and trophic level contribute to the observed MeHg-driven risk patterns. Predatory species like pike exhibited the highest methylmercury burdens: for example, the composite sample of pike from Jugla* Lake had a risk ratio of 3.48 at 300 g/week, indicating that consuming ~300 g of these fish in a week would result in MeHg exposure about 3.5 times the tolerable limit. By comparison, fish groups limited by PFASs were generally those with low mercury levels; their PFAS-based risk ratios were below 1 at 300 g/week but happened to exceed the corresponding (lower) mercury risk ratios for those particular groups. Figure 3 illustrates the relative contributions of MeHg and PFASs to the total risk in each group, highlighting which contaminant is the dominant driver at the 300 g/week scenario.

### 3.2. Nutrient Contributions

**Figure 4:**
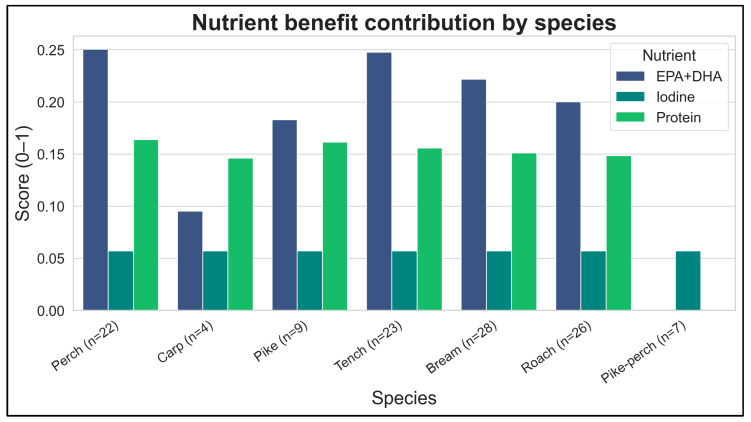
Nutrient contributions at the 300 g/week scenario (70 kg adult). Capped benefit ratios (0–1) are shown for EPA + DHA, protein, and iodine by lake × species group, expressed relative to the weekly reference intakes used in this study; values are capped at 1 to prevent dominance by a single nutrient.

The median nutrient concentrations in each fish group translated into varying weekly contributions to adult nutrient intakes. EPA + DHA provided the most pronounced benefit among the nutrients assessed. At 300 g/week consumption, many groups delivered a large fraction of the 1750 mg/week EPA + DHA target, with fatty fish (e.g., perch, pikeperch) approaching or reaching the full recommended weekly intake of omega-3. Protein contributions were more modest and relatively uniform across groups—typically on the order of 15–25% of the ~406 g/week protein reference for 300 g of fish consumption (reflecting the high protein content of fish muscle, but limited total quantity consumed). Iodine, on the other hand, contributed negligibly in all groups. Most freshwater fish samples had iodine concentrations near or below the detection limit, so after substituting half-LOQ for non-detects, the estimated iodine intake from 1–3 fish meals per week was minimal and almost constant across groups. This interpretation was supported by the LB/MB/UB sensitivity analysis: varying the substitution approach for iodine and Σ4 PFAS did not change the discrete advice category in any stratum (0/30), and changed the limiting hazard in only 1/30 strata (Usma–Roach) (Supplementary Table S6). The iodine component remained small and showed limited discriminatory power across lake × species strata under all substitution variants (Supplementary Table S6). After capping benefit ratios at 1, variability in the nutritional index was driven primarily by EPA + DHA (and secondarily protein), with iodine contributing minimally across strata (Figure 4; Supplementary Table S6).

### 3.3. Integrated Risk-Benefit Outcomes

**Figure 5:**
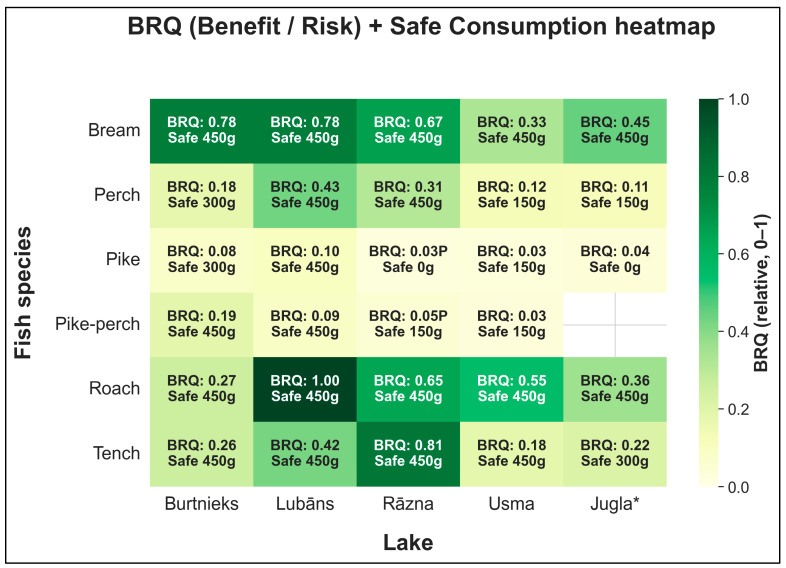
Integrated outputs by lake × species group. Colour shading indicates relative BRQ magnitude (comparative ranking only; no threshold implied). Each cell also reports the discrete safe-consumption category (0/150/300/450 g/week). * *Jugla* indicates trophy-fish sampling in Lake Jugla (minimum fish length 75 cm).

Combining risk and benefit dimensions, we derived safe weekly consumption categories (0/150/300/450 g/week), the limiting hazard (MeHg vs. Σ4 PFAS), and BRQ values for comparative ranking. Integrated outputs for all lake–species strata are summarized in Supplementary Table S3 and visualized in Figure 5. In total, 20/30 strata were categorized as safe up to 450 g/week, 3/30 up to 300 g/week, 5/30 up to 150 g/week, and 2/30 fell into the 0 g/week category (not recommended at 150 g/week). BRQ values are presented for relative comparison only (no threshold implied). BRQ ranking was robust to alternative nutritional index specifications: excluding protein and applying EPA + DHA-emphasized weights yielded high concordance with the main analysis (Spearman ρ = 0.993 and 0.995, respectively; 0/30 strata shifted ≥5 rank positions) (Supplementary Table S7). Figure 5 uses colour shading to indicate relative BRQ magnitude (higher vs. lower within the study), while overlaid text shows the discrete safe-consumption category (0/150/300/450 g/week) and the limiting hazard (MeHg or Σ4 PFAS).

### 3.4. Contaminant Levels and Compliance Context

**Figure 6:**
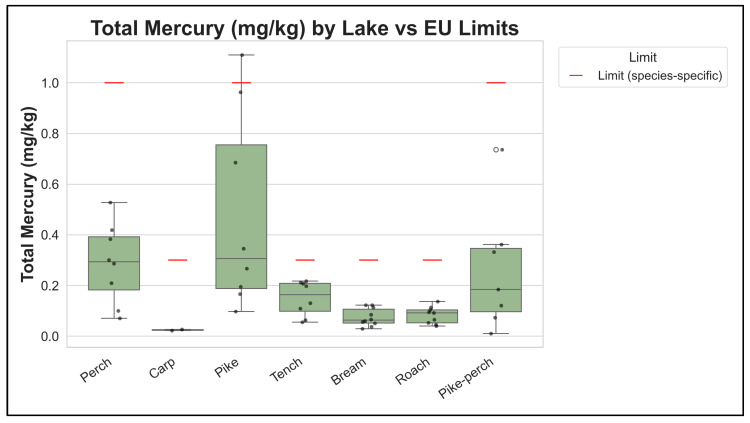
Total mercury concentrations in pooled muscle composites compared with EU maximum levels. Total Hg is shown in mg/kg wet weight by lake × species group; horizontal reference lines indicate EU maximum levels (0.5 and 1.0 mg/kg ww, depending on species category).

It is useful to contextualize these findings with absolute contaminant concentrations relative to regulatory limits. Total mercury concentrations in the pooled fish muscle composites can be compared to European Union maximum levels for mercury in fish (0.5 mg/kg wet weight for most fish and 1.0 mg/kg for specified predatory fish). Figure 6 shows that median total mercury levels were below the relevant EU maximum level across strata, with the highest predatory strata approaching the 1.0 mg/kg level. This compliance perspective complements, but does not replace, the health-based TWI comparison used in the risk ratios, because frequent consumption can exceed TWIs even when maximum levels are not exceeded.

**Figure 7:**
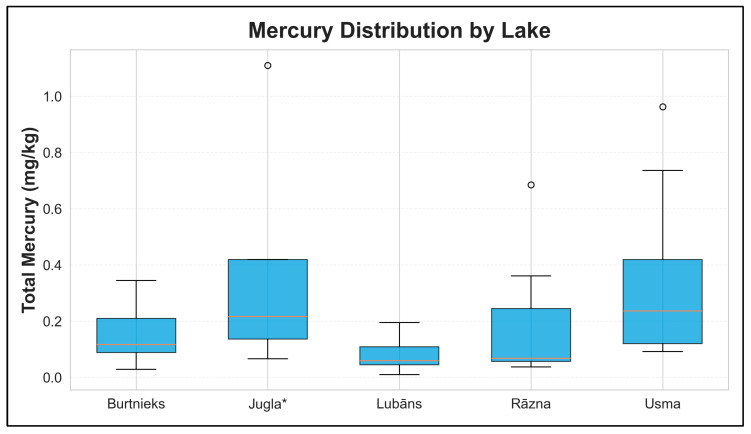
Distribution of total mercury concentrations by lake in pooled muscle composites. Total Hg is shown in mg/kg wet weight; each point represents one pooled composite sample. The plot summarizes between-lake differences and within-lake dispersion across pooled samples. * *Jugla* indicates trophy-fish sampling in Lake Jugla (minimum fish length 75 cm).

Figure 7 summarizes the distribution of total mercury concentrations by lake across pooled samples. Jugla exhibited higher central levels and wider variability than other lakes, consistent with the inclusion of large predatory individuals in the Jugla* stratum. Other lakes generally showed lower mercury levels and narrower dispersion across the pooled composites.

**Figure 8:**
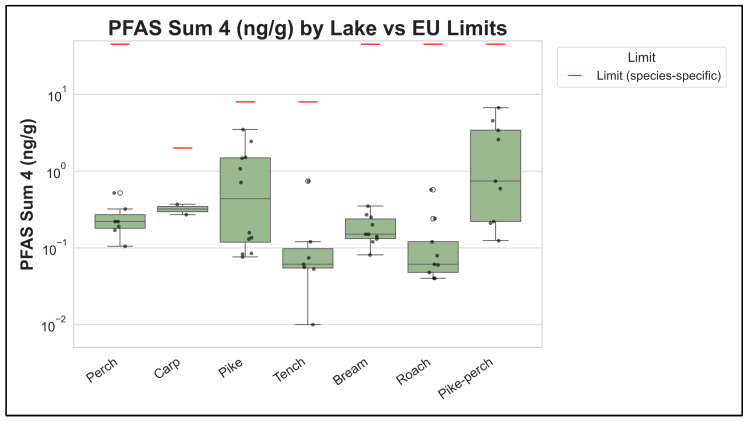
Σ4 PFAS concentrations in pooled muscle composites compared with EU maximum levels. Σ4 PFAS is the sum of PFOS, PFOA, PFNA, and PFHxS (wet-weight basis; units as shown on the axis). The horizontal reference line indicates the EU maximum level for fish meat; the *y*-axis is logarithmic.

For PFASs, the European Commission has established maximum levels in fish meat for the sum of PFOS, PFOA, PFNA, and PFHxS. In our dataset, Σ4 PFAS median concentrations were far below these limits (the highest median Σ4 PFAS was <1 µg/kg), which is also evident in Figure 8 (logarithmic scale). The Supplementary Materials section provides additional details on analytical coverage (Figure S1), concentration distributions (Figures S2–S9), and the full decision matrix (Table S3).

## 4. Discussion

### 4.1. Main Risk Drivers and Implications for Advice

Consistent with global findings for fish consumption risk–benefit analyses, methylmercury was the predominant risk driver in this inland fish dataset [21]. Predatory species, which occupy higher trophic levels, tend to accumulate mercury to levels that can constrain safe intake, whereas PFASs in these samples contributed comparatively less to the estimated risk. The observation that PFASs were the limiting factor in only five out of 30 groups (and even then, not leading to TWI exceedance at 300 g/week) suggests that, at least for the lakes and analytes covered here, mercury is the more critical contaminant for frequent fish consumers to be mindful of. That said, PFAS contamination is highly site-specific; a different set of lakes or downstream of industrial areas could yield a greater PFAS impact. The few cases where Σ4 PFAS “dominated” risk in our results were those with very low mercury levels—for example, a smaller non-predatory fish from a lake with moderate PFAS pollution. Importantly, even in those cases, the PFAS risk ratios remained well below 1.0 at the scenario intakes. In a broader context, PFASs are an emerging food safety concern, and EFSA has noted that parts of the European population exceed the PFAS TWI through combined dietary exposure [7]. Our results highlight that while PFASs currently play a secondary risk role for Latvian lake fish, continued monitoring is needed. PFASs could become more significant if environmental levels rise, or if additional PFASs are considered.

From a consumer advisory perspective, the lake × species resolution of this assessment provides a clear advantage: it enables specific and straightforward consumption recommendations tailored to each situation. Rather than giving the public generic advice (“limit freshwater fish intake to X per week” or “avoid predatory fish”), risk managers can now say, for example, “bream from Lake Y can be eaten up to two times per week, but pike from Lake Z should be limited to once per month,” and so on, based on evidence. Such precise messaging is possible because we identify not only how much fish is safe to eat but also which contaminant is the limiting factor in each case. This approach aligns with calls in the literature to make risk–benefit assessment outputs more practical and actionable for regulators and consumers [21]. By reducing complex data into an intuitive format, a few consumption categories and a named hazard driver, we make it more likely that scientific advice will be adopted in fish consumption guidelines. As noted in Section 2.1 and Section 4.4, Jugla* represents an intentional upper-end (trophy) stratum and should not be generalized to typical-size fish.

### 4.2. Nutritional Benefits in Context

On the benefit side, our results underscore that the primary nutritional value inland fish provides is via their omega-3 fatty acid content. These nutrients are well known to support cardiovascular and neurodevelopmental health, and regular fish consumption—especially oily fish—has been linked to reduced risk of heart disease [22]. In our study, fish groups that were richer in EPA + DHA clearly stood out with higher nutritional index scores. The protein contribution of fish, while important as a general dietary component, was less variable among groups and thus less influential in distinguishing one fish from another in risk–benefit terms. Most fish provide a lean protein source; in a typical 150 g portion of fish, one might get roughly 30–35 g of protein, which is beneficial but not uniquely high compared to other protein foods. Iodine, as discussed, was practically a non-factor here due to low levels in freshwater fish. Marine fish and seafood are typically much higher in iodine content than freshwater fish [22], because seawater contains far more iodine that enters the marine food web.

It is worth noting that the risk–benefit calculation capped each nutrient’s contribution at 100% of daily needs. This was a conservative choice taken to avoid, for example, oily fish scoring extremely high just because they exceed the omega-3 recommendation by a large margin. Beyond a certain point, additional omega-3 intake may have diminished returns for health, so our capping approach reflects that. The outcome of this is that several fish reached the maximum benefit for EPA + DHA at the higher consumption scenarios. This is a positive from a nutritional standpoint—it means moderate amounts of fish can supply all the recommended omega-3 for the week. Accordingly, the benefit profile in this dataset is driven largely by omega-3 content, while iodine contributes minimally in freshwater fish relative to marine sources [23]. Overall, our integrated assessment reinforces the idea that promoting fish intake should be accompanied by an emphasis on species (or sources) that deliver the greatest nutritional upsides with the least contaminant risk.

### 4.3. Communicating Safe Consumption Guidance

One of the goals of this pilot was to produce outputs that are readily interpretable by non-specialists. We chose to express the advice in terms of weekly consumption categories that correspond to common meal frequencies. This categorical approach (e.g., “up to 1 serving per week” or “up to 3 servings per week”) is consistent with how agencies communicate fish advisories (e.g., meal-frequency groupings in FDA guidance) [24]. Our scheme of 0, 150, 300, 450 g/week is analogous to “0, 1, 2, or 3 servings per week,” providing a simple phrase that can be communicated (assuming ~150 g is a serving). By integrating the contaminant driver into the message, risk managers can also convey why a particular limit is in place—for example, a recommendation might read: “Perch from Lake Burtnieks—safe for 2 meals per week (limit driven by mercury)**.**” This one-liner contains what a consumer or angler needs to know in practical terms, without overwhelming them with numbers.

Of course, simplifying continuous data into broad categories does entail the loss of information. In our results, a fish categorized as “150 g/week” safe could have an actual safe consumption limit anywhere from just above 150 g to just below 300 g. We opted to round down to the nearest category to be precautionary. Stakeholders using this information should recognize that these categories are screening-level guides. They are very useful for comparing and prioritizing—for example, identifying which fish are low risk (450 g/week category) versus which are high risk (150 or 0 g/week category)—but they are not meant to be precise prescriptions. Individual risk can vary (some fish meals may be larger or smaller; individuals have different body weights and sensitivities, etc.). Therefore, we advise that the numerical results (continuous safe grams per week) be made available in technical appendices for transparency, even if the public-facing guidance sticks to simple categories. In short, the discrete categories serve communication, while the underlying numbers remain for reference.

Despite these caveats, the benefit–risk quotient and safe intake outputs offer a clear improvement over more generic advice. They allow risk managers to update guidance as new data comes in, and to target risk mitigation efforts. For example, if certain lake–species combos are consistently in the “0” category due to mercury, authorities might consider posting notices at those lakes or promoting the consumption of alternative species. Conversely, if some local fish are in the “450 g/week, low risk” category and nutritious, that could be a positive message to encourage their consumption as part of a healthy diet. This balanced messaging—eat more of these fish, eat less of those fish—is exactly what an integrated risk–benefit framework aims to enable. It avoids the pitfall of blanket warnings that might inadvertently discourage all fish consumption. Ultimately, our results can feed directly into public health communication materials like the FDA’s fish advice chart but tailored to Latvian inland fish and local consumption patterns.

### 4.4. Limitations and Future Perspectives

Being a pilot study, this assessment has several limitations that warrant discussion. First, the analysis is deterministic and uses median values from pooled samples. This means we did not explicitly account for variability or uncertainty in the contaminant and nutrient levels. Individual fish (and individual consumers’ habits) vary, so a certain percentage of fish might have higher mercury than our median, potentially making the true “safe” consumption lower for unlucky consumers who consistently catch or eat those high-mercury individuals. The pooling strategy and use of medians smooth out those highs and lows, effectively focusing on an “average” exposure scenario. Accordingly, the outputs are most appropriately interpreted at the lake × species stratum level as central-tendency guidance and should not be read as estimates of individual-level exposure distributions for frequent consumers. This is appropriate for a conservative screening, but it does not capture worst-case exposures. To quantify how much this central-estimate choice affects the practical decision outputs, we re-ran the calculations using the mean concentration and, as a conservative check, the maximum pooled-composite concentration within each lake × species group (Table S5). Using the mean instead of the median did not change the discrete advice category (0/150/300/450 g/week) or the limiting hazard for any of the 30 lake × species strata. Using the maximum pooled-composite concentration led to more restrictive advice categories in 6/30 strata and changed the limiting hazard in 2/30 strata, while the Jugla* strata were unchanged under these checks. Because iodine measurements were frequently <LOQ, the iodine component contributes minimally and provides limited differentiation across strata; the LB/MB/UB sensitivity confirms that this censoring has negligible impact on the practical consumption guidance categories (Supplementary Table S6). Seasonality is another representativeness constraint. Samples were collected during two periods (Autumn 2024 and Summer 2025), but the pooled design and differences in lake/species composition between seasons limit formal attribution. An exploratory pooled-sample comparison (Supplementary Table S4) suggested no statistically significant difference for total Hg (*p* = 0.123), while Σ4 PFAS (*p* = 0.0439) and EPA + DHA (*p* = 0.0244) differed between seasons, with fat and protein showing no clear differences. These results should be interpreted as indicative rather than conclusive; repeated within-stratum sampling would be required to quantify seasonal effects on the final guidance outputs. Future assessments should incorporate variability—for instance, using probabilistic models or at least examining percentile concentrations—especially if moving toward setting regulatory advice or if sensitive subpopulations (pregnant women, children) are considered.

Second, there were data gaps in our hazard coverage. PFAS measurements were not available for two strata (Rāzna–pike and Rāzna–pikeperch) due to analytical limitations; these were assessed using mercury only and explicitly flagged as PFAS data gaps (Supplementary Table S3). If those groups in fact had undetected PFASs, their true safe intake might be lower. More broadly, this pilot hazard set does not fully represent the spectrum of chemicals that may be relevant in some inland fisheries (e.g., PCBs/dioxins or other legacy contaminants), and inclusion of such compounds could tighten consumption guidance for specific lake × species strata, particularly predatory fish where co-occurrence is plausible. The framework is modular: additional hazards can be incorporated by adding further risk-ratio components (RRi) alongside MeHg and Σ4 PFAS when occurrence data become available. Expanding PFAS monitoring to cover all relevant fish is a clear next step, as PFASs remain a dynamic and emerging risk area. PFASs can originate from multiple source types, and catchment-level source attribution was outside the scope of this pilot. Latvian inland-water monitoring using composite freshwater fish samples suggests that PFAS contamination reflects multiple sources and is generally lower than in heavily industrialized regions, although localized elevated areas cannot be excluded [11]. In that Latvian dataset, PFOA showed low detection frequency and concentrations (45% detection; mean 0.01 ng/g ww), whereas PFOS dominated the PFAS profile (~60% of total selected PFAS) [11], which is consistent with the low PFOA contribution observed in fish muscle. EFSA has explicitly recommended gathering more occurrence data on PFASs in a wide range of foods (including freshwater fish) to support dietary exposure assessments [7]. In line with that, ongoing surveillance of PFASs in inland waters is important. Moreover, other contaminants not assessed here (such as legacy organochlorine pesticides, PCBs, or dioxins) could be present in some lake fish. We focused on mercury and PFASs as priority hazards, but a more comprehensive risk assessment would eventually need to include additional chemical residues that are known to occur regionally. A further limitation is that MeHg was estimated from total Hg using a fixed fraction (0.95×), and potential species- or lake-specific variation in MeHg/total Hg ratios could affect risk estimates; dedicated speciation measurements would be needed to confirm and refine this parameter for Latvian inland fish. That said, many of those other contaminants tend to co-occur with mercury in predatory fish (e.g., pike can also accumulate PCBs and dioxins), so mercury may act as a proxy for a general “chemical load” to some extent. Still, our framework is modular and can be extended with new analytes—either adding new risk components or refining the existing ones.

On the benefit side, only three components were quantified in this pilot (EPA + DHA, iodine, protein). Other nutrients often associated with fish consumption benefits (e.g., selenium and vitamin D) were not measured here and could change the relative benefit profiles and BRQ ranking for some strata, especially for fattier fish. Importantly, the hazard-based safe-consumption outputs (g/week and category) depend on health-based guidance values for contaminants and would not change by adding benefit components; rather, additional nutrients would primarily refine the comparative benefit dimension and integrated ranking.

Another limitation is the reliance on guideline reference values and the absence of a common health metric to directly compare benefits and risks (we used separate indices and then the BRQ ratio). Some risk–benefit analyses in the literature attempt to compute a single net health impact (e.g., in DALYs), which can directly address net benefit versus harm. We did not adopt such an endpoint-based health-metric approach in this pilot because it would substantially increase complexity and require additional assumptions and inputs (e.g., endpoint selection, dose–response functions, baseline disease rates, subgroup stratification, and uncertainty propagation). Instead, we used a transparent heuristic approach (TWI-based risk ratios and %RDI-based benefit scores) and interpreted the BRQ strictly as a comparative ranking indicator rather than an absolute risk metric. Consequently, we do not propose BRQ thresholds for “favourable” versus “unfavourable” profiles. Importantly, the stability of BRQ ranking under minimal alternative assumptions is supported by the sensitivity analyses for left-censored handling and nutritional index construction (Supplementary Tables S6 and S7). EFSA’s risk–benefit assessment guidance and ongoing methodological developments provide a clear pathway for future work to extend this pilot towards more formal endpoint-based health impact metrics, if needed [25].

The framework is presented as a transferable decision-support approach, but the numerical outputs are context-specific. The methodological structure (deterministic risk ratios based on health-based guidance values, transparent benefit scoring, and identification of safe-consumption categories and limiting hazards) can be applied in other regions. However, transfer requires re-parameterization using locally representative occurrence data for the relevant species and edible tissues (including associated LOQs), together with region-appropriate hazard and nutrient priorities and consumption assumptions. With these inputs, the same modular calculations can produce locally tailored guidance while maintaining methodological consistency.

Finally, the strengths and opportunities of this work should be highlighted alongside its limitations. Methodologically, a key strength is the transparency and updateability of the framework. All calculations are straightforward, and if new data come in (e.g., a new lake sampled, or repeated sampling in these lakes in a future year), the model can be rerun easily to refresh the advice. The use of clear rules (median, deterministic scenarios, capped benefits, etc.) means the process can be understood by decision-makers without advanced statistical training. This transparency is crucial for trust and uptake of recommendations. Another strength is the direct practicality of the output: we have essentially created a decision table that can guide fish consumption in a nuanced way. This addresses the initial objective of moving beyond one-size-fits-all messaging. From a risk management standpoint, this pilot can inform monitoring priorities—for example, it pinpointed Jugla pike as a high-risk case, suggesting that particular attention be paid to mercury in that lake’s food chain. It also identified certain fish as low risk, which could be promoted as safer choices. In terms of next steps for risk management, authorities could use these results to update consumer advisory pamphlets, angler education programs, or dietary guidelines to incorporate lake-specific advice. On the monitoring side, repeating this assessment periodically (e.g., every few years) would be valuable to track trends. If, say, mercury levels decrease due to environmental measures, or if PFAS levels increase due to emerging pollution sources, the guidance can be adjusted. Continuous monitoring of contaminant levels in these inland fish—as recommended by other researchers for public health protection [26]—will ensure that consumption advice remains science-based and protective.

## 5. Conclusions

This study presents a deterministic pilot risk–benefit assessment of Latvian inland lake fish using pooled, lake × species-stratified composites to generate practical consumption guidance. Across 30 strata, methylmercury was the dominant constraint on safe intake for most predatory fish, whereas Σ4 PFAS limited only a small subset of low-mercury strata. Most lake–species combinations were categorized as safe up to 450 g/week, while a small number of predatory strata required stricter limits. On the benefit side, EPA + DHA provided the primary differentiating signal between strata, whereas iodine contributed minimally due to frequent left-censoring. The framework is transparent and modular and can be updated as monitoring coverage expands (additional lakes/species, contaminants, and nutrients). Key limitations are the pooled and median-based central tendency design and the partial hazard/benefit set; future work should broaden analyte coverage and incorporate variability (e.g., repeated within-stratum sampling and probabilistic modelling) where consumer advice for sensitive subpopulations is required.

## Data Availability

Data supporting the findings of this study are available from the authors upon reasonable request. Primary analytical data are not publicly archived at the time of publication due to institutional data management constraints and because the dataset forms part of an ongoing monitoring and reporting workflow. Any processed summary tables and derived metrics used in the manuscript can be shared to enable verification of the reported results.

## Supplementary Materials

The following supporting information can be downloaded at: https://www.mdpi.com/article/10.3390/foods15050901/s1, Additional supporting information (full dataset and appendices with Tables S1–S7 and Figures S1–S11) is available in the online version of this article.

## Abbreviations

The following abbreviations are used in this manuscript:

Abbreviation	Meaning
BR	benefit ratio
BRQ	benefit–risk quotient
BW	body weight
C18	octadecylsilane (C18) stationary phase
CONTAM	EFSA Panel on Contaminants in the Food Chain
DHA	docosahexaenoic acid
EFSA	European Food Safety Authority
EPA	eicosapentaenoic acid
FAME	fatty acid methyl esters
GC-FID	gas chromatography with flame ionization detection
H2O2	hydrogen peroxide
HNO3	nitric acid
HPLC	high-performance liquid chromatography
ICP-MS	inductively coupled plasma mass spectrometry
LC	liquid chromatography
LOQ	limit of quantification
MeHg	methylmercury
PFASs	per- and polyfluoroalkyl substances
PFNA	perfluorononanoic acid
PFOA	perfluorooctanoic acid
PFOS	perfluorooctane sulfonate
PRM	parallel reaction monitoring
PUFA	polyunsaturated fatty acids
RBA	risk–benefit assessment
RR	risk ratio
SPE	solid-phase extraction
TMAH	tetramethylammonium hydroxide
TWI	tolerable weekly intake
Σ4 PFAS	sum of PFOS, PFOA, PFNA, and PFHxS
