# Supplementary material for: Deterministic Pilot Risk–Benefit Assessment of Latvian Inland Fish: Safe Weekly Consumption Guidance
Source: Foods. 2026 Mar 5;15(5):901. doi: 10.3390/foods15050901 (PMC12984848; doi:10.3390/foods15050901)
Supplement: Supplementary file 1 [file foods-15-00901-s001.zip › foods-4146455-supplementary.pdf]

Supplementary

This Supplementary contains additional context figures and tables that support the main Results/Discussion.

- Important note on iodine: In the pooled fillet dataset used here, iodine measurements were all left-censored (<LOQ) and therefore collapse to a constant value after LIMS handling. For this reason iodine is not included in the seasonal comparison plots.

Table S1. Data completeness by category

Category	Analyses intended, n	Analyses performed, n	Completeness
Fat	67	67	100.0%
Fatty acids	67	61	91.0%
Iodine	63	59	93.7%
Metals	67	67	100.0%
PFAS	67	56	83.6%
Protein	67	67	100.0%

Reminder on Table S1 denominators: the *Analyses intended*/*Analyses performed* counts come from the project's planned analytical completeness file for the core RBA subset (n=67 pooled fillet composites).

Table S2. Fillet sample count by lake × species (pooled)

	Sample count, n					
**Species**	**Burtnieks**	**Lubāns**	**Rāzna**	**Usma**	**Jugla***	**Total**
Bream	6	9	5	6	2	28
Carp	0	4	0	0	0	4
Perch	6	3	5	6	2	22
Pike	2	2	1	2	2	9
Pike-perch	2	2	1	2	0	7
Roach	6	6	5	6	3	26
Tench	6	3	5	6	3	23

	Sample count, n					
Species	Burtnieks	Lubāns	Rāzna	Usma	Jugla*	Total
Total	28	29	22	28	12	119

* Lake Jugla was sampled using a trophy-fish design (minimum fish length 75 cm).

Table S3. Lake × species recommended safe weekly consumption category and limiting hazard

Lake	Species	Safe (g/week)	Limiting factor	BRQ	Hg_total (mg/kg)	Σ4 PFAS (ng/g)	EPA+DHA (mg/kg)
Burtnieks	Perch	300	MeHg	0.179	0.255	0.312	1226
Burtnieks	Pike	300	MeHg	0.083	0.221	0.106	NA
Burtnieks	Tench	450	MeHg	0.264	0.171	0.372	1251
Burtnieks	Bream	450	Σ4 PFAS	0.781	0.043	0.160	943
Burtnieks	Roach	450	MeHg	0.267	0.104	0.130	318
Burtnieks	Pike-perch	450	MeHg	0.189	0.097	0.172	NA
Jugla	Perch	150	MeHg	0.115	0.419	0.170	1276
Jugla	Pike	0	MeHg	0.039	1.110	0.130	1067
Jugla	Tench	300	MeHg	0.217	0.217	0.053	1332
Jugla	Bream	450	Σ4 PFAS	0.450	0.066	0.350	1431
Jugla	Roach	450	MeHg	0.359	0.136	0.060	1447
Lubāns	Perch	450	MeHg	0.433	0.100	0.220	1126
Lubāns	Carp	450	Σ4 PFAS	0.310	0.024	0.320	555
Lubāns	Pike	450	MeHg	0.101	0.180	0.084	NA
Lubāns	Tench	450	MeHg	0.425	0.109	0.120	1216

Lake	Species	Safe (g/week)	Limiting factor	BRQ	Hg_total (mg/kg)	Σ4 PFAS (ng/g)	EPA+DHA (mg/kg)
Lubāns	Bream	450	MeHg	0.783	0.059	0.140	1311
Lubāns	Roach	450	MeHg	1.005	0.049	0.100	1452
Lubāns	Pike-perch	450	Σ4 PFAS	0.087	0.095	0.665	NA
Rāzna	Perch	450	MeHg	0.312	0.178	0.220	1730
Rāzna	Pike	0	MeHg	0.027	0.685	NA	NA
Rāzna	Tench	450	MeHg	0.816	0.059	0.065	1380
Rāzna	Bream	450	MeHg	0.671	0.080	0.115	1671
Rāzna	Roach	450	MeHg	0.648	0.053	0.041	658
Rāzna	Pike-perch	150	MeHg	0.051	0.361	NA	NA
Usma	Perch	150	MeHg	0.125	0.455	0.255	1803
Usma	Pike	150	MeHg	0.030	0.615	0.158	NA
Usma	Tench	450	MeHg	0.184	0.202	0.061	862
Usma	Bream	450	MeHg	0.330	0.117	0.260	933
Usma	Roach	450	Σ4 PFAS	0.554	0.098	0.309	1827
Usma	Pike-perch	150	MeHg	0.034	0.534	0.210	NA

Table S4. Exploratory season comparison (pooled fillet samples)

Season comparisons are exploratory because pooled sampling and lake/species composition can confound differences. **Summer corresponds to 2025 sampling and Autumn corresponds to 2024 sampling**. P-values are from a two-sided Mann–Whitney U test.

Analyte	n (Summer)	n (Autumn)	Median (Summer)	Median (Autumn)	p-value
Hg_total (mg/kg)	24	28	0.153	0.0955	0.123
Σ4 PFAS (ng/g)	18	28	0.112	0.164	0.0439
EPA+DHA (mg/kg)	25	36	861	1.37e+03	0.0244
Fat (%)	30	37	0.87	1.03	0.65
Protein (%)	30	37	20.2	20.1	0.7

Table S5. Sensitivity analysis of the deterministic central estimate (median vs mean vs maximum pooled-composite). The table summarizes how the discrete advice category (0/150/300/450 g/week) and the limiting hazard (MeHg vs Σ4 PFAS) change when the lake × species central estimate is computed as the mean or as the maximum pooled-composite concentration (conservative check), relative to the main median-based analysis. Flags indicate whether the advice category or limiting hazard differs from the main analysis.

Lake	Species	Main category	Mean category	Max category	Main limiting hazard	Mean limiting hazard	Max limiting hazard	Category changed (mean)	Category changed (max)	Limiting hazard changed (mean)	Limiting hazard changed (max)
Burtnieks	Perch	300	300	300	MeHg	MeHg	MeHg	No	No	No	No
Burtnieks	Pike	300	300	150	MeHg	MeHg	MeHg	No	Yes	No	No
Burtnieks	Tench	450	450	300	MeHg	MeHg	Σ4 PFAS	No	Yes	No	Yes
Burtnieks	Bream	450	450	450	Σ4 PFAS	Σ4 PFAS	Σ4 PFAS	No	No	No	No
Burtnieks	Roach	450	450	450	MeHg	MeHg	MeHg	No	No	No	No
Burtnieks	Pike-perch	450	450	450	MeHg	MeHg	MeHg	No	No	No	No
Jugla*	Perch	150	150	150	MeHg	MeHg	MeHg	No	No	No	No
Jugla*	Pike	0	0	0	MeHg	MeHg	MeHg	No	No	No	No
Jugla*	Tench	300	300	300	MeHg	MeHg	MeHg	No	No	No	No
Jugla*	Bream	450	450	450	Σ4 PFAS	Σ4 PFAS	Σ4 PFAS	No	No	No	No
Jugla*	Roach	450	450	450	MeHg	MeHg	MeHg	No	No	No	No
Lubāns	Perch	450	450	450	MeHg	MeHg	MeHg	No	No	No	No
Lubāns	Carp	450	450	450	Σ4 PFAS	Σ4 PFAS	Σ4 PFAS	No	No	No	No
Lubāns	Pike	450	450	450	MeHg	MeHg	MeHg	No	No	No	No
Lubāns	Tench	450	450	450	MeHg	MeHg	MeHg	No	No	No	No
Lubāns	Bream	450	450	450	MeHg	MeHg	MeHg	No	No	No	No
Lubāns	Roach	450	450	450	MeHg	MeHg	MeHg	No	No	No	No

Lake	Species	Main category	Mean category	Max category	Main limiting hazard	Mean limiting hazard	Max limiting hazard	Category changed (mean)	Category changed (max)	Limiting hazard changed (mean)	Limiting hazard changed (max)
Lubāns	Pike-perch	450	450	300	Σ4 PFAS	Σ4 PFAS	Σ4 PFAS	No	Yes	No	No
Rāzna	Perch	450	450	300	MeHg	MeHg	MeHg	No	Yes	No	No
Rāzna	Pike	0	0	0	MeHg	MeHg	MeHg	No	No	No	No
Rāzna	Tench	450	450	450	MeHg	MeHg	MeHg	No	No	No	No
Rāzna	Bream	450	450	450	MeHg	MeHg	MeHg	No	No	No	No
Rāzna	Roach	450	450	450	MeHg	MeHg	MeHg	No	No	No	No
Rāzna	Pike-perch	150	150	150	MeHg	MeHg	MeHg	No	No	No	No
Usma	Perch	150	150	150	MeHg	MeHg	MeHg	No	No	No	No
Usma	Pike	150	150	0	MeHg	MeHg	MeHg	No	Yes	No	No
Usma	Tench	450	450	450	MeHg	MeHg	MeHg	No	No	No	No
Usma	Bream	450	450	450	MeHg	MeHg	MeHg	No	No	No	No
Usma	Roach	450	450	450	MeHg	MeHg	Σ4 PFAS	No	No	No	Yes
Usma	Pike-perch	150	150	0	MeHg	MeHg	MeHg	No	Yes	No	No

Table S6. Sensitivity of left-censored substitution (LB/MB/UB) for iodine and Σ4 PFAS. This table summarizes the impact of lower-bound (LB = 0), middle-bound (MB = LOQ/2), and upper-bound (UB = LOQ) substitution for left-censored values on the iodine component, nutrient score, BRQ ranking, Σ4 PFAS risk ratios, and the primary decision outputs (advice category; limiting hazard).

Across LB/MB/UB variants, the advice category remained unchanged for all strata; the limiting hazard changed only for the Usma–Roach stratum.

Lake	Species	Iodine component (LB/MB/UB)	Nutrient score (LB/MB/UB)	BRQ rank (LB/MB/UB)	Σ4 PFAS RR (LB/MB/UB)	Category changed?	Limiting hazard changed?
Burtnieks	Perch	0.000/0.057/0.114	0.123/0.142/0.161	18/20/20	0.301/0.306/0.311	No	No
Burtnieks	Pike	0.000/0.057/0.114	0.000/0.057/0.114	26/25/23	0.103/0.111/0.118	No	No
Burtnieks	Tench	0.000/0.057/0.114	0.122/0.142/0.161	15/16/17	0.400/0.407/0.414	No	No
Burtnieks	Bream	0.000/0.057/0.114	0.103/0.122/0.141	4/4/3	0.154/0.156/0.159	No	No
Burtnieks	Roach	0.000/0.057/0.114	0.067/0.086/0.105	16/15/16	0.123/0.135/0.147	No	No
Burtnieks	Pike-perch	0.000/0.057/0.114	0.000/0.057/0.114	26/18/13	0.169/0.179/0.189	No	No
Jugla*	Perch	0.000/0.057/0.114	0.132/0.151/0.170	21/22/25	0.162/0.167/0.171	No	No
Jugla*	Pike	0.000/0.057/0.114	0.115/0.134/0.153	22/27/30	0.126/0.135/0.145	No	No
Jugla*	Tench	0.000/0.057/0.114	0.129/0.148/0.167	17/17/18	0.052/0.061/0.071	No	No
Jugla*	Bream	0.000/0.057/0.114	0.135/0.154/0.173	8/8/8	0.343/0.343/0.343	No	No
Jugla*	Roach	0.000/0.057/0.114	0.134/0.153/0.172	11/11/11	0.058/0.068/0.078	No	No
Lubāns	Perch	0.000/0.057/0.114	0.117/0.136/0.155	9/9/9	0.216/0.221/0.226	No	No
Lubāns	Carp	0.000/0.057/0.114	0.080/0.099/0.119	14/14/14	0.313/0.320/0.328	No	No

Lake	Species	Iodine component (LB/MB/UB)	Nutrient score (LB/MB/UB)	BRQ rank (LB/MB/UB)	Σ4 PFAS RR (LB/MB/UB)	Category changed?	Limiting hazard changed?
Lubāns	Pike	0.000/0.057/0.114	0.000/0.057/0.114	26/23/21	0.082/0.092/0.101	No	No
Lubāns	Tench	0.000/0.057/0.114	0.126/0.145/0.164	10/10/10	0.113/0.118/0.123	No	No
Lubāns	Bream	0.000/0.057/0.114	0.126/0.145/0.164	3/3/4	0.137/0.142/0.147	No	No
Lubāns	Roach	0.000/0.057/0.114	0.134/0.153/0.172	1/1/1	0.094/0.102/0.109	No	No
Lubāns	Pike-perch	0.000/0.057/0.114	0.000/0.057/0.114	26/24/22	0.652/0.657/0.662	No	No
Rāzna	Perch	0.000/0.057/0.114	0.155/0.174/0.193	13/13/15	0.212/0.212/0.212	No	No
Rāzna	Pike	0.000/0.057/0.114	0.000/0.057/0.114	26/30/29	NA/NA/NA	No	No
Rāzna	Tench	0.000/0.057/0.114	0.132/0.151/0.170	2/2/2	0.063/0.071/0.078	No	No
Rāzna	Bream	0.000/0.057/0.114	0.148/0.167/0.186	5/5/6	0.112/0.117/0.122	No	No
Rāzna	Roach	0.000/0.057/0.114	0.088/0.107/0.126	6/6/5	0.046/0.056/0.066	No	No
Rāzna	Pike-perch	0.000/0.057/0.114	0.000/0.057/0.114	26/26/26	NA/NA/NA	No	No
Usma	Perch	0.000/0.057/0.114	0.159/0.178/0.197	20/21/24	0.245/0.253/0.260	No	No
Usma	Pike	0.000/0.057/0.114	0.000/0.057/0.114	26/29/28	0.155/0.160/0.165	No	No
Usma	Tench	0.000/0.057/0.114	0.098/0.117/0.136	19/19/19	0.059/0.069/0.079	No	No
Usma	Bream	0.000/0.057/0.114	0.102/0.121/0.141	12/12/12	0.249/0.254/0.259	No	No
Usma	Roach	0.000/0.057/0.114	0.152/0.172/0.191	7/7/7	0.302/0.310/0.317	No	Yes
Usma	Pike-perch	0.000/0.057/0.114	0.000/0.057/0.114	26/28/27	0.206/0.211/0.216	No	No

Table S7. Sensitivity of BRQ ranking to nutritional index construction (300 g/week). This table compares BRQ ranks under the base nutritional index (equal weighting of capped EPA+DHA, iodine, and protein components) to two minimal alternatives: excluding protein and applying an EPA+DHA-emphasized weighting scheme. Rank differences are shown for each lake × species stratum.

Lake	Species	Rank (base)	Rank (no protein)	Rank (weighted)	Δrank (no protein)	Δrank (weighted)
Burtnieks	Perch	20	19	18	1	2
Burtnieks	Pike	25	25	25	0	0
Burtnieks	Tench	16	14	15	2	1
Burtnieks	Bream	3	4	4	1	1
Burtnieks	Roach	15	18	17	3	2
Burtnieks	Pike-perch	18	17	20	1	2
Jugla*	Perch	22	22	22	0	0
Jugla*	Pike	27	27	27	0	0
Jugla*	Tench	17	16	16	1	1
Jugla*	Bream	8	8	8	0	0
Jugla*	Roach	11	11	11	0	0
Lubāns	Perch	9	9	9	0	0
Lubāns	Carp	13	15	14	2	1

Lake	Species	Rank (base)	Rank (no protein)	Rank (weighted)	Δrank (no protein)	Δrank (weighted)
Lubāns	Pike	23	23	23	0	0
Lubāns	Tench	10	10	10	0	0
Lubāns	Bream	4	3	3	1	1
Lubāns	Roach	1	1	1	0	0
Lubāns	Pike-perch	24	24	24	0	0
Rāzna	Perch	14	12	12	2	2
Rāzna	Pike	30	30	30	0	0
Rāzna	Tench	2	2	2	0	0
Rāzna	Bream	5	5	5	0	0
Rāzna	Roach	6	7	7	1	1
Rāzna	Pike-perch	26	26	26	0	0
Usma	Perch	21	21	21	0	0
Usma	Pike	29	29	29	0	0
Usma	Tench	19	20	19	1	0
Usma	Bream	12	13	13	1	1
Usma	Roach	7	6	6	1	1
Usma	Pike-perch	28	28	28	0	0

Figure S1. Analytical coverage vs planned (pooled fillet samples)

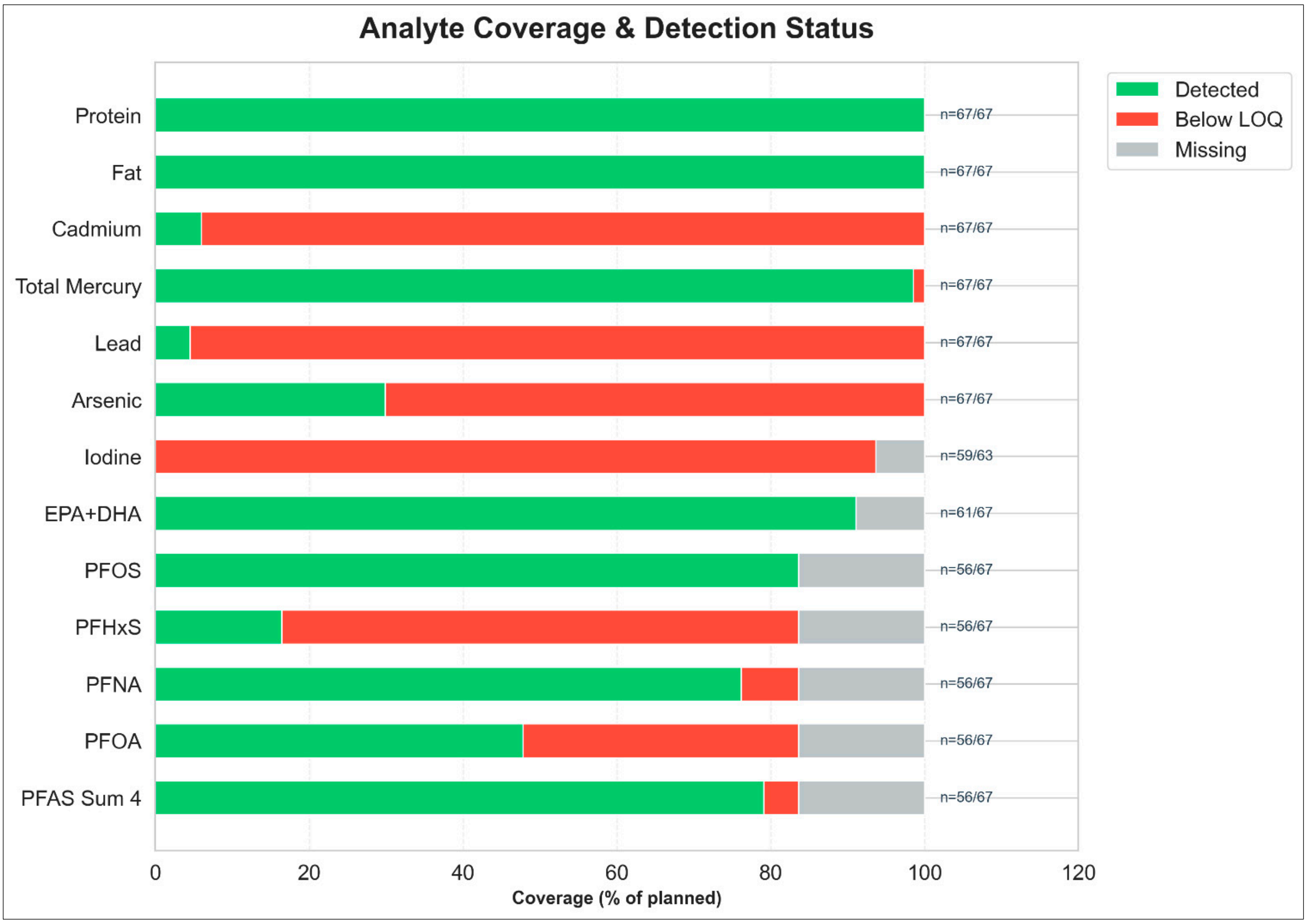

Figure S2 provides the univariate distribution of detected mercury values in pooled fillet samples. The vertical reference lines (mean/median where applicable) help communicate both the central tendency and skewness, which is important because deterministic risk ratios are median-based while consumer exposure concerns often relate to upper tails.

Figure S2. Mercury distribution (detected values)

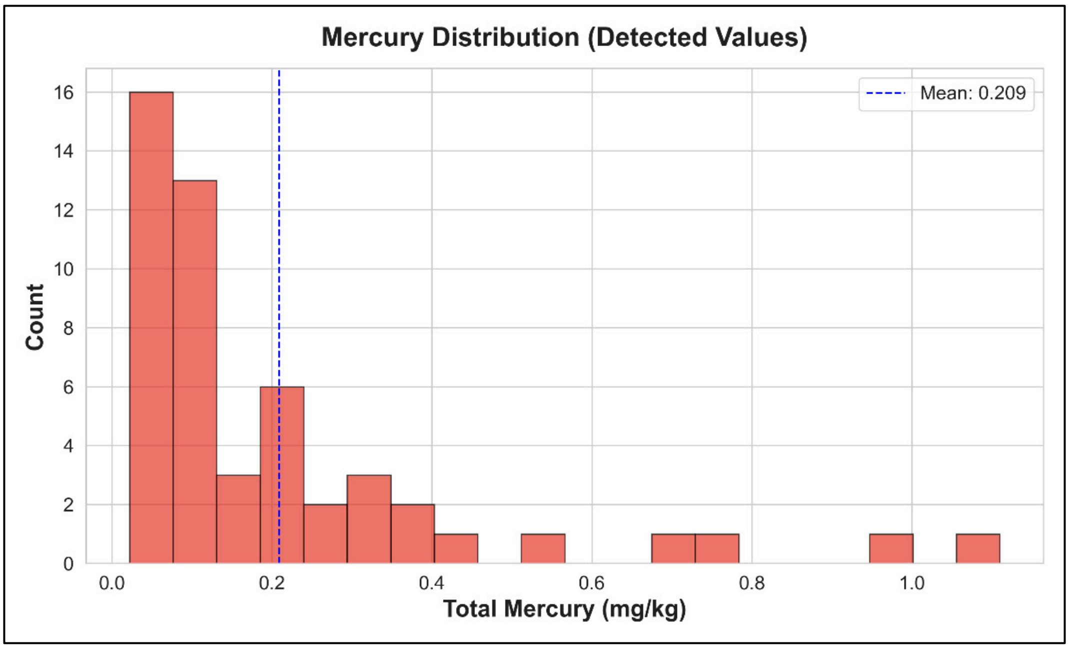

Figure S3 summarizes protein content across pooled fillet samples. Protein is expected to be relatively stable across fish, so this figure primarily serves as a data-quality and plausibility check for the nutrition side of the assessment.

Figure S3. Protein distribution

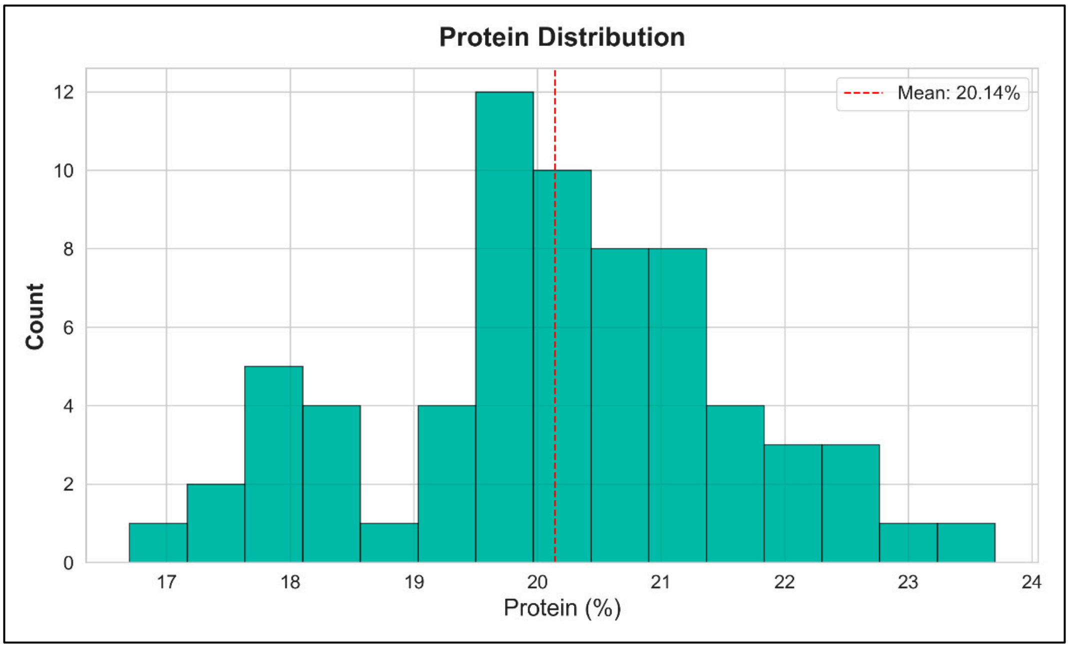

Figure S4 breaks down protein distributions by species, allowing a quick check for systematic species differences and potential outliers that could disproportionately influence mean-based summaries.

Figure S4. Protein distribution by species

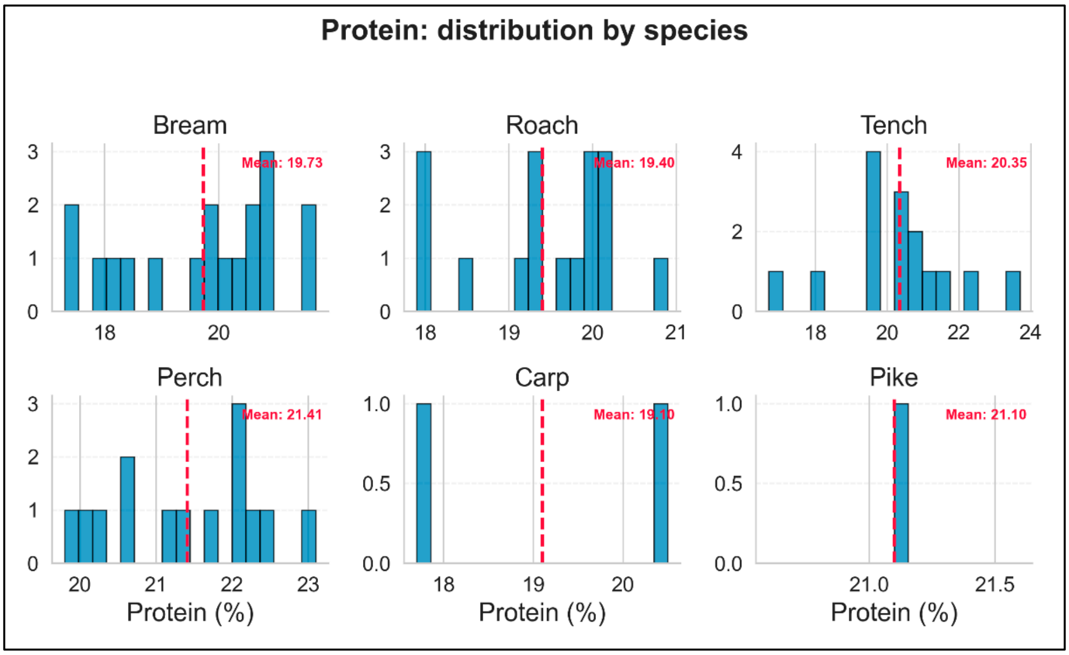

Figure S5 shows fat content by species, which is relevant because EPA+DHA is derived from fatty acid composition and fat content. Species differences in fat can therefore propagate into differences in EPA+DHA on a wet-weight basis.

Figure S5. Fat distribution by species

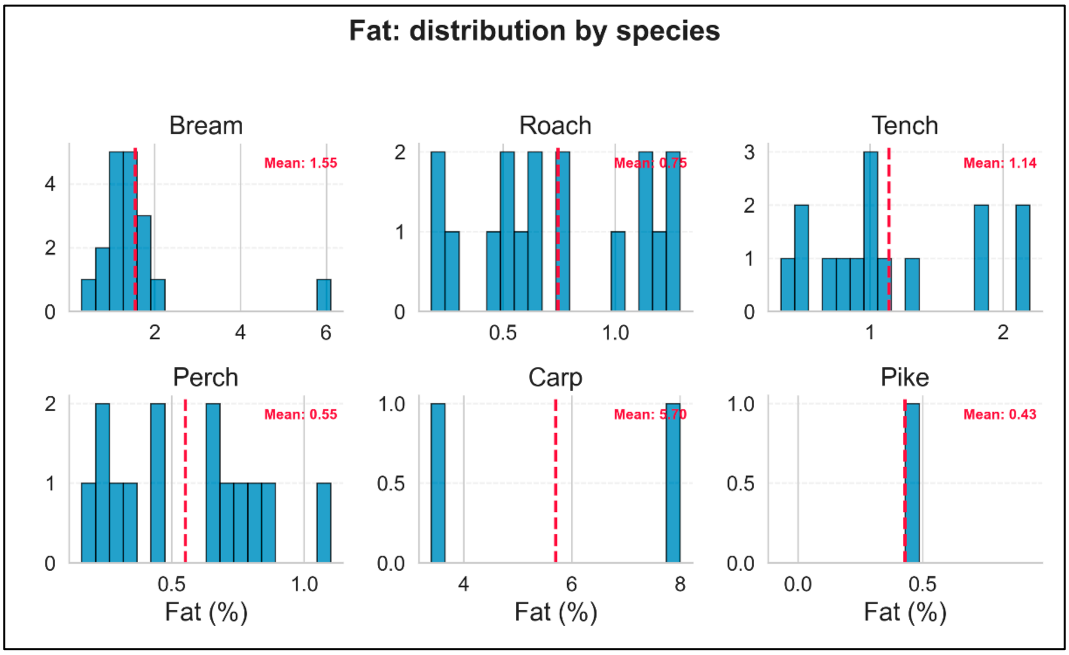

Figure S6 focuses on EPA+DHA by species. This directly supports interpretation of Figure 3 because EPA+DHA is typically the dominant differentiating nutrient component between species.

Figure S6. EPA+DHA distribution by species

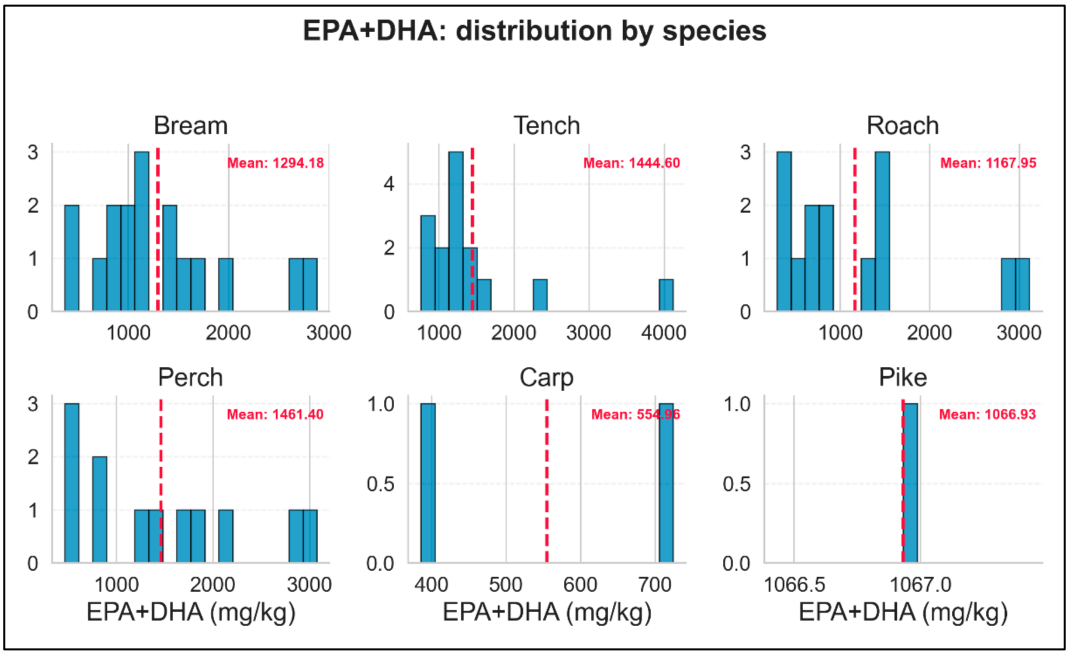

Figure S7 provides the overall EPA+DHA distribution across pooled fillet samples. This is useful for assessing whether the benefit signal is driven by broad shifts across groups or by a smaller number of high-benefit groups.

Figure S7. EPA+DHA distribution

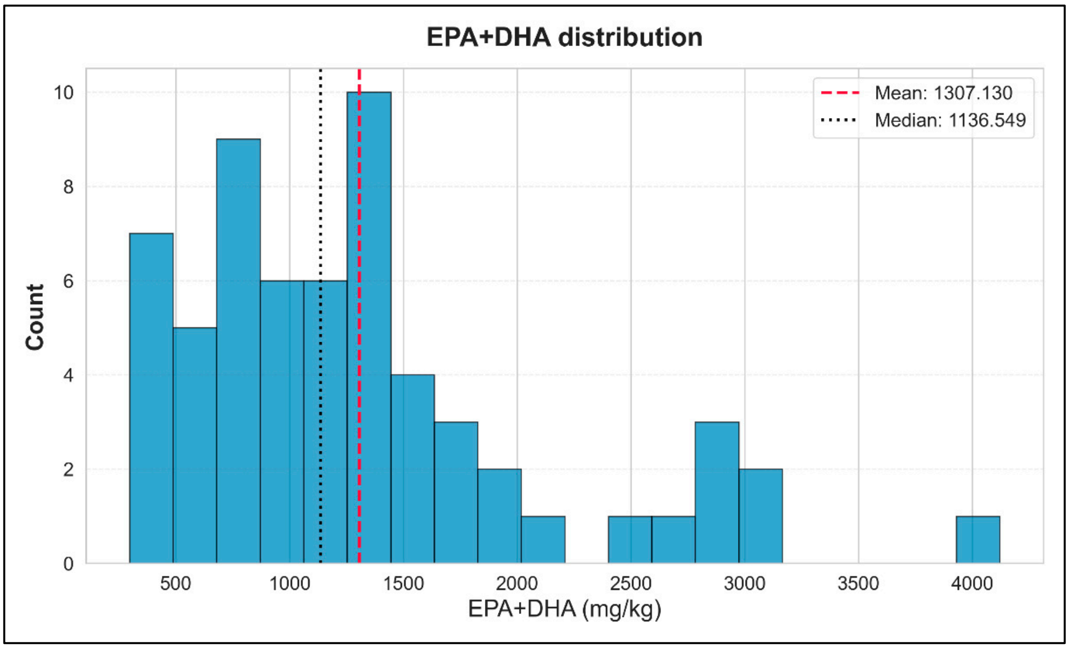

Figure S8 provides the overall Σ4 PFAS distribution on a log scale, which is appropriate for right-skewed concentration data. The mean/median markers help interpret whether central tendency is dominated by a few higher values.

Figure S8. Σ4 PFAS distribution (log scale)

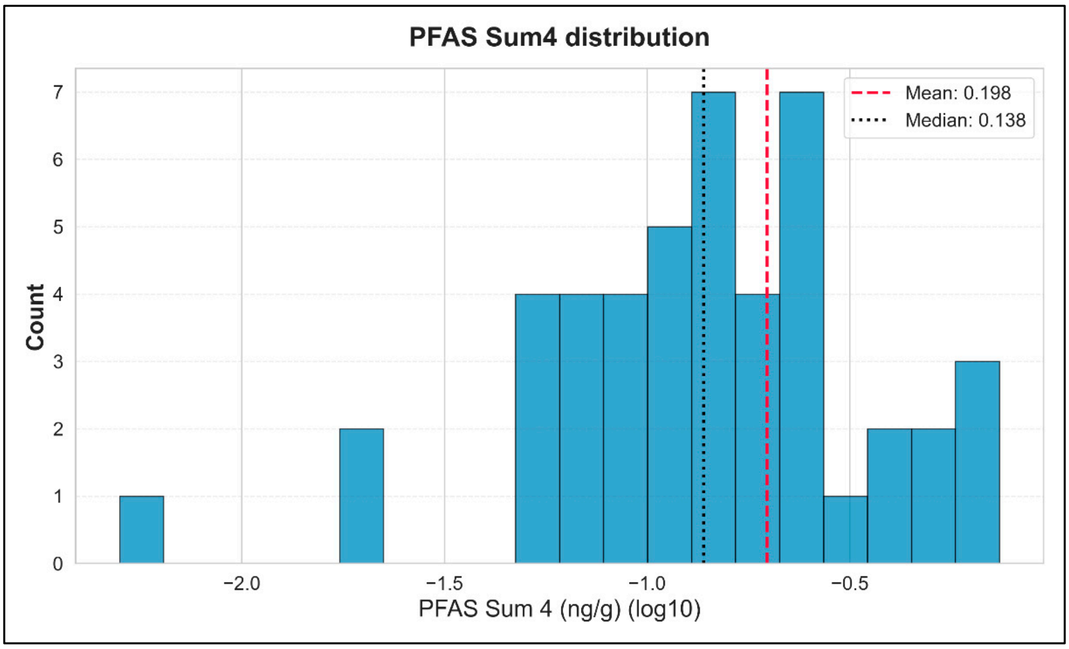

Figure S9 reports the pooled sampling effort by lake (fillet/muscle only). Counts refer to the number of composite (pooled) fillet samples per lake. This is a key representativeness context figure: lakes with fewer pooled samples provide weaker support for precise lake-level comparisons.

Figure S9. Pooled sample counts by lake

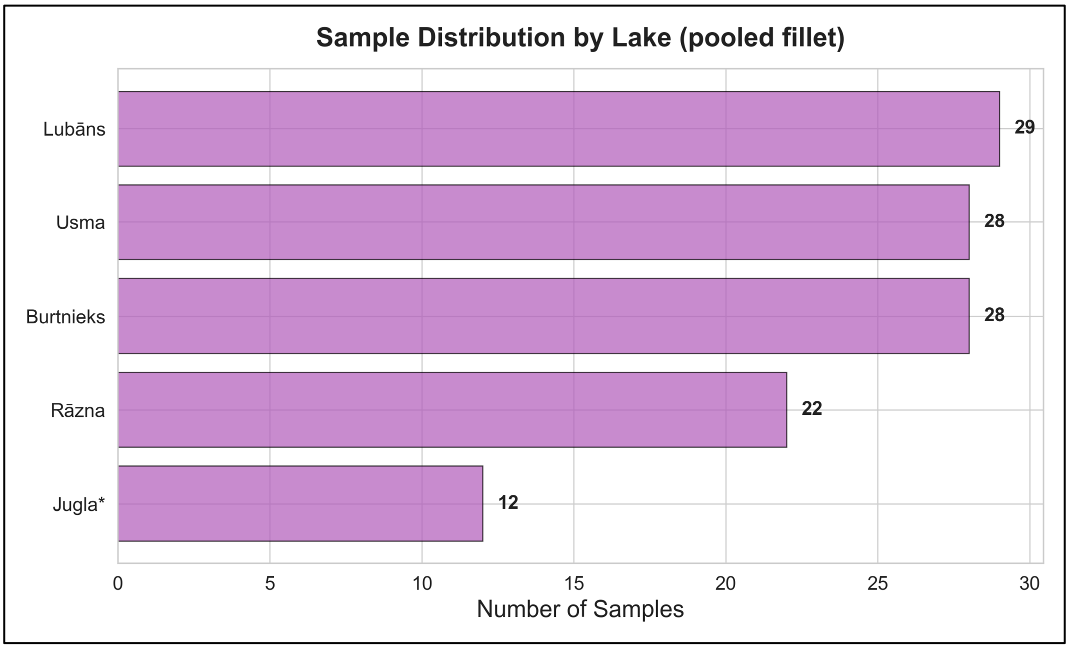

Figure S10 provides an exploratory comparison between summer and autumn pooled fillet samples for key contaminants and nutrients. Summer corresponds to 2025 sampling and Autumn corresponds to 2024 sampling. Because the dataset is pooled and seasonal sampling is not perfectly balanced across lakes and species, these comparisons should be interpreted cautiously (season and composition may be confounded).

Figure S10. Season comparison (pooled fillet samples)

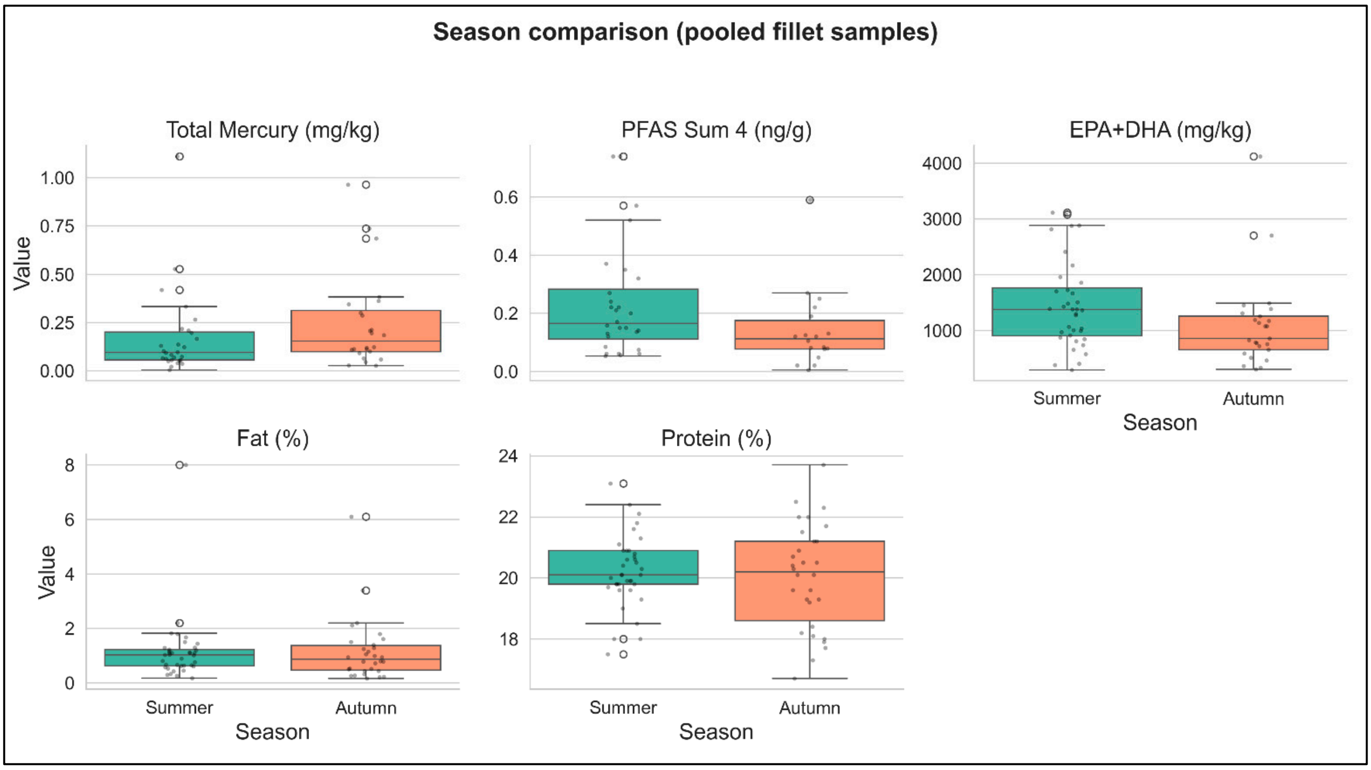

Figure S11 compares the distribution of maximum risk ratios between Jugla *groups and other lakes at the 300 g/week scenario. This provides a simple sensitivity check given the representativeness note for Jugla (trophy-fish focus). In the pooled fillet dataset, Jugla contributes n=12 composites and has 33% predatory-species composites, compared with 32% in other lakes (n=107).*

Figure S11. Sensitivity to Jugla groups (300 g/week)

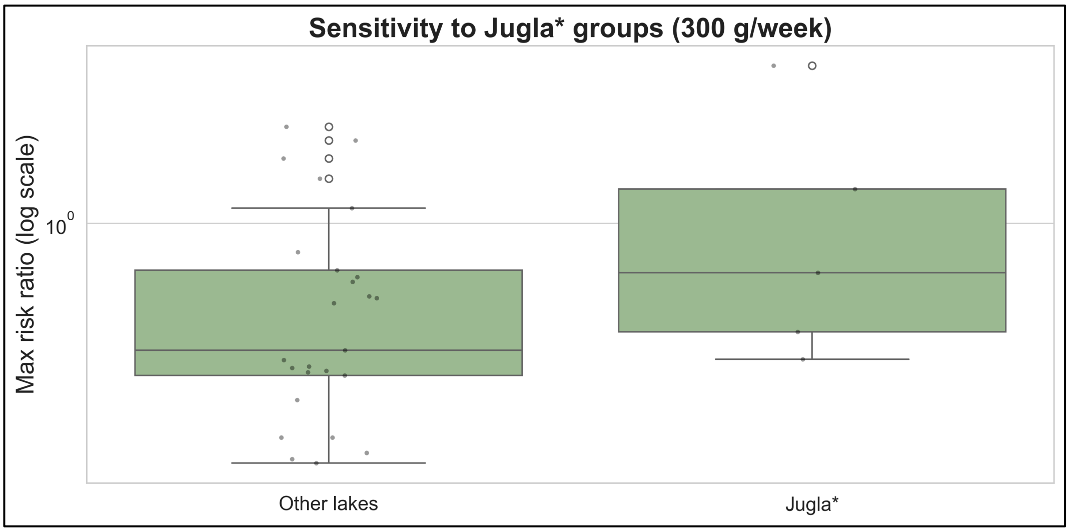
Sensitivity to Jugla* groups (300 g/week)
Max risk ratio (log scale)
10^0
Other lakes
Jugla*
